# Phosphorus availability drives mycorrhiza induced resistance in tomato

**DOI:** 10.3389/fpls.2022.1060926

**Published:** 2022-12-19

**Authors:** Laura Dejana, Beatriz Ramírez-Serrano, Javier Rivero, Jordi Gamir, Juan A. López-Ráez, María J. Pozo

**Affiliations:** ^1^ Department of Soil Microbiology and Symbiotic Systems, Estación Experimental del Zaidín, Consejo Superior de Investigaciones Científicas (CSIC), Granada, Spain; ^2^ Institut de Recherche sur la Biologie de l’Insecte (IRBI), UMR 7261, /Universite de Tours Centre National de la Recherche Scientifique (CNRS), Tours, France; ^3^ Plant Immunity and Biochemistry Group, Department of Biology Biochemistry and Natural Sciences, Universitat Jaume I, Avd. Vicente Sos Baynat s/n, Castellón, Spain

**Keywords:** DAMPs (damage-associated molecular patterns), defense priming, jasmonate signalling, plant immunity, plant nutrition, oligogalacturonides (OGs), herbivory, pathogen

## Abstract

Arbuscular mycorrhizal (AM) symbiosis can provide multiple benefits to the host plant, including improved nutrition and protection against biotic stress. Mycorrhiza induced resistance (MIR) against pathogens and insect herbivores has been reported in different plant systems, but nutrient availability may influence the outcome of the interaction. Phosphorus (P) is a key nutrient for plants and insects, but also a regulatory factor for AM establishment and functioning. However, little is known about how AM symbiosis and P interact to regulate plant resistance to pests. Here, using the tomato-*Funneliformis mosseae* mycorrhizal system, we analyzed the effect of moderate differences in P fertilization on plant and pest performance, and on MIR against biotic stressors including the fungal pathogen *Botrytis cinerea* and the insect herbivore *Spodoperta exigua.* P fertilization impacted plant nutritional value, plant defenses, disease development and caterpillar survival, but these effects were modulated by the mycorrhizal status of the plant. Enhanced resistance of *F. mosseae*-inoculated plants against *B. cinerea* and *S. exigua* depended on P availability, as no protection was observed under the most P-limiting conditions. MIR was not directly explained by changes in the plant nutritional status nor to basal differences in defense-related phytohormones. Analysis of early plant defense responses to the damage associated molecules oligogalacturonides showed primed transcriptional activation of plant defenses occurring at intermediate P levels, but not under severe P limitation. The results show that P influences mycorrhizal priming of plant defenses and the resulting induced-resistance is dependent on P availability, and suggest that mycorrhiza fine-tunes the plant growth vs defense prioritization depending on P availability. Our results highlight how MIR is context dependent, thus unravel molecular mechanism based on plant defence in will contribute to improve the efficacy of mycorrhizal inoculants in crop protection.

## Introduction

The development of sustainable technologies for crop management aiming to reduce the use of fertilizers and pesticides is an ongoing global challenge in agriculture (Pretty and Bharucha, 2018). In this scenario, the potential of beneficial symbiotic microbes to improve plant nutrition and stress resistance/tolerance is well established (Berendsen et al., 2012; Pieterse et al., 2014; Pozo et al, 2020). However, their performance under agronomic conditions is not optimal, as the outcomes of plant-microbe interactions are highly context-dependent and may vary according to the plant growing conditions (van der Heyde et al., 2017; Lee Díaz et al., 2021). Among the beneficial organisms used as inoculants in agriculture, fungi, and particularly arbuscular mycorrhizal fungi (AMF), are receiving increasing interest (Pozo et al., 2021). These soil-borne microorganisms, belonging to the phylum *Glomeromycota*, are widespread in natural and agricultural ecosystems and colonize the roots of more than 80% of terrestrial plant species, including major crops (Smith and Smith, 2011). This mutualistic association, known as mycorrhiza, has major benefits for both partners: AMF receive plant organic carbon in the form of carbohydrates and lipids (Keymer et al., 2017; Salmeron-Santiago et al, 2021). In return, AMF improve water and nutrients uptake by the plant, especially phosphorus (P) (Chen et al., 2018; Ferrol et al, 2019). In addition, they also increase plant phenotypic and metabolic plasticity to cope with biotic and abiotic stressors (Rivero et al., 2018; Sanchez-Bel et al, 2018; Campo et al, 2020; Mitra et al., 2021; Rivero et al., 2021; Pozo de la Hoz et al., 2021).

AM symbiosis usually renders the plant more resistant to certain soil-borne and aboveground pathogens and chewing herbivores, as shown in multiple systems (Jung et al., 2012; Song et al., 2013; Song et al., 2015; Selvaraj and Thangavel, 2021; Dowarah et al., 2022). This Mycorrhiza-Induced Resistance (MIR) is related to an improved ability of mycorrhizal plants to trigger defense responses upon challenge, a cost-efficient strategy known as defense priming (Pozo and Azcón-Aguilar, 2007; Martinez-Medina et al., 2016; Mauch-Mani et al., 2017). Priming is a common mechanism during induced systemic resistance triggered by beneficial microbes, consisting of a stronger and faster activation of plant defense mechanisms, usually dependent on jasmonate signaling (Pieterse et al., 2014; Mora-Romero et al., 2014; Gruden et al., 2020). Despite priming of plant defenses in response to pathogens and herbivores in mycorrhizal plants is well documented (Campos-Soriano et al., 2012; Song et al., 2013; Song et al., 2015; Sanchez-Bel et al., 2016; Fiorilli et al., 2018; Schoenherr et al., 2019; Sanmartín et al., 2020; Rivero et al., 2021; Manresa-Grao et al., 2022) consequences of AM symbiosis for insect herbivores are complex to predict. Indeed, the nutritional benefits of the symbiosis may increase food availability or improve the diet quality for the pest, depending on the fungal and insect identity and growing conditions (Koricheva et al., 2009; Frew and Price, 2019). Thus, the interaction outcome results from the interplay of opposite effects: nutritional improvement, benefiting the herbivore, and defense priming, potentially reducing herbivore performance (Pozo et al., 2020). Indeed, plant-microbe-insect interactions are very complex, with plants coordinating their responses according to multiple external and internal cues through a precise regulation orchestrated by phytohormone networks (Pozo et al., 2015; Gruden et al., 2020).

Many biotic and abiotic factors influence mycorrhizal colonization (Diagne et al., 2020; Mitra et al., 2021). Indeed, the plant-AMF interaction is finely regulated by several factors, including the plant and fungal genotypes and environmental conditions, with nutrients availability as one of the most influential aspects (Pozo et al., 2015; Lee Díaz et al., 2021). P is a central regulator of mycorrhizal establishment, root colonization and fungal growth within the host plant. Thus, the abuse of P fertilizers inhibits the establishment of mycorrhizal symbiosis in different plants (Smith and Smith, 2011; Smith et al., 2011; Higo et al., 2020). P, an essential nutrient for plants, is a non-renewable resource and poorly available in the field, being a limiting factor for plant growth (Wang et al., 2021). This element is taken up from the soil in the form of inorganic P by the roots (Rouached et al., 2010), and its deficiency triggers important changes in plant physiology and biochemistry including changes in plant growth, metabolism, hormonal balance, gene expression and root architecture. P deficiency further promotes the production of the phytohormones strigolactones, accumulation of anthocyanins and other phenolic compounds (Ha and Tran, 2014; Vysotskaya et al., 2016; Marro et al., 2022).

In agriculture, chemical and organic fertilizers are strongly and widely applied to solve P soil deficiency, causing contamination of aquifers and altering plant interactions with beneficial microbes, including AMF (Smith and Smith, 2011; Cordell and White, 2015). P levels also impact plant interactions with other organisms by altering plant tissues’ nutritional value for organisms feeding on them (pathogens and herbivores) and by modulating plant defenses (Breuillin et al, 2010; Castrillo et al, 2017; Chan et al., 2021). The role of P in plant immunity is currently under intense scrutiny, and the data reveal a very complex scenario (Chan et al., 2021; Qu et al., 2021; Val-Torregrosa et al, 2022). The phosphate starvation response in plants has been suggested to increase plant resistance to necrotrophic pathogens and leaf chewing herbivores in several plant species (Arabidopsis, tomato, tobacco) pointing to a positive cross-talk between the JA- and P- starvation signaling pathways that activates plant immunity (Khan et al., 2016). However, opposite results have been also found in different plant species facing different aggressors (Campo et al., 2020; Val-Torregrosa et al, 2022). It should be noted that most basic studies dealing with the molecular mechanisms mediating P effects on plant immunity compare very contrasting P levels, usually far from those used in agricultural settings.

Despite the increasing number of studies addressing P influence on plant defenses, only a few have investigated the relationship between P and MIR, suggesting a complex interaction with contrasting results (Wang et al., 2020; Qu et al., 2021). While evidence of the role of P in regulating mycorrhizal colonization, plant growth and immunity responses exist, the interplay among the different effects and mechanisms behind this interplay remain poorly studied (Wang et al., 2020; Qu et al., 2021). Our study aims to elucidate whether the mycorrhizal effect on tomato resistance to biotic stressors is regulated by P availability. For that, we evaluated the impact of different P fertilization regimes on tomato growth, mycorrhizal colonization and herbivore resistance using the tomato/*Funneliformis mosseae* system and the necrotrophic pathogen *Botrytis cinerea* and the generalist chewing herbivore *Spodoptera exigua*. We found that P availability strongly influences plant growth and resistance to biotic stresses, and that these effects are modulated by the AM symbiosis, showing that MIR is indeed dependent on P availability. By analyzing plant nutrients, defense-related phytohormones and transcriptional regulation of defenses in response to the damage-associated signals oligogalacturonides (OGs), we aimed to uncover the mechanistic basis of the impact of P availability on MIR. This knowledge will contribute to improve AMF application and management for sustainable crop protection.

## Material and methods

### Biological material and mycorrhizal inoculation

The AMF *Funneliformis mosseae* BEG12 (Young, 2015) obtained from the International Bank of Glomeromycota (http://www.i-beg.eu), was maintained at the EEZ-CSIC greenhouse as open-pot cultures of *Trifolium repens* mixed with *Sorghum vulgare* plants using vermiculite-sepiolite substrate. The inoculum consisted in the substrate containing colonized root fragments, fungal mycelia and spores.

*Botrytis cinerea* was cultivated in potato dextrose agar plates, supplemented with freeze-dried tomato leaves. Three weeks later, *B. cinerea* spores were collected from plates in 0.5X potato dextrose broth as previously described (Sanmartín et al., 2020).

*Spodoptera exigua* (Lepidoptera: Noctuidae) eggs were provided by Dr. S. Herrero lab (ERI-BIOTECMED, Universitat de Valencia, Spain). Larvae were reared on artificial diet (Greene et al., 1976) at 25°C with 16:8h light:dark regime and 70% relative humidity until L2-L3 larval stage, for their application.

Tomato seeds (*Solanum lycopersicum* L. cv. Moneymaker) were surface disinfected by immersion in 4% NaHClO (10 min), rinsed thoroughly with sterile water and incubated for 10 days in an open container with sterile vermiculite at 25°C. Plantlets were transferred to 100 mL pots containing a sterile sand:vermiculite (1:1) mixture. Pots for mycorrhizal treatments were inoculated by adding 10% (v/v) *F. mosseae* inoculum. All plants, including non-inoculated ones, received a 3 ml aliquot of a filtrate (<20 μm) of the inoculum, in order to provide the general microbial population but free of AMF spores.

### Experimental design

Different experiments were carried out to elucidate the effect of P nutrition on plant performance and herbivore resistance in mycorrhizal and non-mycorrhizal plants. All experiments included tomato plants inoculated with *F. mosseae* (Fm) or not (Nm). We performed a first screening using three P concentrations: 0.3 mM, 0.6 mM and 1.0 mM (10 plants per treatment). For this, plants were watered with Hewitt nutrient solution (Hewitt, 1996), modified in the P (H_2_NaPO4) concentration as described in **Table S1**. Plants were harvested after 8 weeks of growth and the performance of *S. exigua* fed on detached leaves was evaluated (see herbivore bioassays below). Plant biomass, mycorrhizal colonization, plant nutrients, anthocyanin content and phytohormone levels were also determined.

Based on the results obtained, 0.3 or 0.7 mM P concentrations were selected for follow-up experiments. In a second experiment, we assessed P effects on *Botrytis cinerea* lesion development through a detached leaf assay as described below, and we evaluated the performance of *S. exigua* larvae directly fed on Fm and Nm plants (whole plant bioassay). Six-weeks-old plants were infested with *S. exigua* larvae and caterpillar performance (weight, survival and pupation) was steadily monitored for 3 weeks (9 plants per treatment). Finally, early plant defense responses were compared in mycorrhizal and non-mycorrhizal plants growing at these two P levels (0.3 and 0.7 mM; 6 plants per treatment). For that, we assessed the plant response to damage by using oligogalacturonides (OGs), well characterized damage-associated molecular patterns (DAMPs) (see Plant treatment with oligogalacturonides below). The levels of defense-related phytohormones and defense-related gene expression were determined 6 hours post treatment (hpt) as described below. Thus, the factors considered in this study were M: mycorrhizal inoculation (levels: Nm, Fm), P: P fertilization regimes (levels 0.3, 0.7 and 1.0 mM), and OG: Oligogalacturonide treatment (levels: -OG, +OG).

### Plant growing conditions

For the different experiments, plants were randomly distributed and grown in a greenhouse at 24/16°C with a 16/8 h diurnal photoperiod and 70% humidity. Plants were watered twice a week with half strength Hewitt nutrient solution (Hewitt, 1996) modified in the P content as described above, and water was supplied as needed. Upon harvesting shoots and roots, fresh weight was determined and the material was immediately frozen in liquid N and stored at -80°C for further analyses. An aliquot of each individual root system was preserved for mycorrhizal quantification.

### *Botrytis cinerea* infection

The fourth leaf of tomato plants was detached for pathogen bioassays. Pathogen infection was performed by applying 10 μL drops containing a conidia suspension (1 × 10^6^ spores/ml) to the detached leaves. One leaf per plant was inoculated by adding two drops per leaflet, five leaflets per leaf, with a total of 90 lesions. The infected leaves were maintained in 15cm Petri dishes on wet filter paper and incubated in a phytotron chamber at 80% of humidity and 22°C. Necrotic lesions were measured 3 days post-inoculation.

### Herbivore performance bioassays

Insect performance was evaluated in two different bioassays, using detached leaves or whole plants. For the detached leaves assay, two second-instar *S. exigua* larvae were placed on one detached leaf of each tomato plant, placed on wet filter paper in 15 cm Petri dishes. Plates were then incubated in a phytotron at 26-24°C, 16:8h day/night and 60% relative humidity. Larval mortality and weight was evaluated every 2 days for 8 days.

For whole plants assay, two second-instar larvae were placed in a leaflet of the fourth true leaf of each tomato plant, using clip-cages to avoid their escape, as described in Rivero et al. (2021). Every two days, clip-cages were moved to new fresh leaflets and caterpillar biomass, mortality and pupation were monitored.

### Plant treatment with oligogalacturonides

For the analysis of early plant responses, the damage associated molecules oligogalacturonides (OGs) were used to elicit plant defense responses. The OGs (DP 10-15) were prepared as described in Benedetti et al. (2017) and provided by Dr. De Lorenzo lab (Department of Biology and Biotechnology “Charles Darwin” BBCD, La Sapienza University, Rome, Italy).

Six weeks post AMF inoculation tomato plants grown at 0.3 or 0.7 mM P fertilization regimes were treated with an aqueous solution of OGs (50 μg/ml in milliQ water) as described in Gamir et al. (2020). The fourth true leaf of each plant was sprayed with the OG solution or milliQ water for control plants using an aerograph until running off. Treated leaves were harvested after 6 hours to study early plant defense responses, as this time was the most appropriate to identify changes in hormone contents and in the expression levels of OG responsive, defense related (Gamir et al., 2020).

### Determination of mycorrhizal colonization

AM colonization was measured after clearing washed roots in KOH (10%) and staining fungal structures with 5% ink in 2% acetic acid (Vierheilig et al., 2005). The percentage of total root length colonized by *F. mosseae* was estimated according to the gridline intersection method (Giovannetti and Mosse, 1980) using a BOECO zoom stereo microscope Model BST-606.

### Anthocyanin content

Anthocyanin content, as P starvation indicator, was evaluated by adapting the protocol established in Rabino and Mancinelli (1986). Frozen leaves were grinded and lyophilized, and 5 mg aliquot of dry tissue was used per sample. The pigments were extracted by shaking the plant freeze-dried leaf powder in acidic (1% HCI, w/v) methanol in dark overnight. Extracts were centrifuged for 10 min at 13000 rpm. The amount of anthocyanin was calculated measuring absorbance at 530 and 657 nm of crude extract, using the formula A530 - 0.25 A657 to compensate for the contribution of chlorophyll and its degradation products to the absorption at 530 nm. Six independent biological replicates were analyzed per treatment.

### Determination of mineral nutrients in leaves

Nutrient analyses were performed at the Technical Services of the Estación Experimental del Zaidín (CSIC). Frozen leaves were grinded and lyophilized, and 50-100 mg aliquot of dry tissue was used per sample. The concentration of P and micronutrients were determined after acid digestion of samples, by inductively coupled plasma optical emission spectrometry (ICP‐OES; Varian ICP 720‐ES). Total C and N contents were analyzed using an Elemental Analyzer (LECO TruSpec CN), according to standard procedures. Six independent biological replicates were analyzed per treatment.

### Targeted hormonal extraction and quantification

Hormone extraction was performed from freeze-dried powdered plant leaves as described in Sanchez-Bel et al. (2016). Six independent biological replicates per treatment were analyzed. Briefly, 30 mg of plant dry tissue was extracted with 1 ml of H_2_O:MeOH (9:1) containing 0.001% of HCOOH and 100 ng/ml of internal standards. After different centrifugations and resuspensions, an aliquot of the extract was injected into an Acquity Ultra Performance Liquid Chromatography system (UPLC) (Waters, Mildford, MA, USA). Hormones were chromatographically separated using an HPLC Kinetex C18 analytical column (Phenomenex) connected to a triple quadrupole mass spectrometer (TQD, Waters, Manchester, UK). The chromatographic and mass spectrometry conditions were those used by Gamir et al. (2012). Hormone quantification (ng/g dry weight) was performed using calibration curves with each pure chemical standard. The plant hormones abscisic acid (ABA), indolacetic acid (IAA), jasmonic acid (JA), its precursor (+)-12-oxo-phytodienoic acid (OPDA) and salicylic acid (SA) were determined.

### Analysis of gene expression by qPCR

The expression of marker genes from different metabolic pathways was analyzed by real time quantitative PCR (qPCR). Six independent biological replicates per treatment were used. Total RNA from tomato leaves was extracted and treated with DNase using the Direct-zol RNA MiniPrep kit (Zymo Research). Subsequently, the RNA was purified through a column using the RNA Clean and Concentrator-5 kit (Zymo Research), and stored at -80°C until use. The first-strand cDNA was synthesized with 1 μg of purified total RNA using the iScript cDNA Synthesis kit (Bio-Rad). All kits were used according to the manufacturer’s suggested protocols.

The expression of three different housekeeping genes, actin (Solyc03g078400), elongation factor 1‐α (Solyc06g005060) and β‐tubulin (Solyc04g081490) was measured to find the optimal normalization gene, using the Normfinder software (https://moma.dk/normfinder-software) (Andersen et al., 2004). According to the results, expression values were normalized using the housekeeping gene β-tubulin and relative quantification of specific mRNA levels was performed using the comparative 2–Δ (ΔCt) method (Livak and Schmittgen, 2001). The sequences of the specific primers used are shown in **Table S2**.

### Statistical analyses

All statistical analyses (multi-way ANOVAs and *post hoc* tests applied when appropriated, as indicated in the corresponding figure legends) were conducted using Statgraphics Plus 3.1 (Rockville, MD, USA) or ‘R’ software v.3.5.2 (R Development Core Team). Figures were obtained using *ggplot2* R package (Wickham et al, 2016). Treatment effects on larval survival were assessed by comparing the survival curves using the Kaplan-Meier estimator (Kaplan and Meier, 1958). Survival distribution comparison between treatments was performed using the non-parametric Logrank test (Mantel-Cox). Survival analyses were performed using *survival* and *survminer* R packages. Model validations were performed using Shapiro-Wilk and Levene’s tests.

## Results

### P fertilization levels impact mycorrhizal colonization, plant growth and herbivore performance in tomato

**Figure 1:**
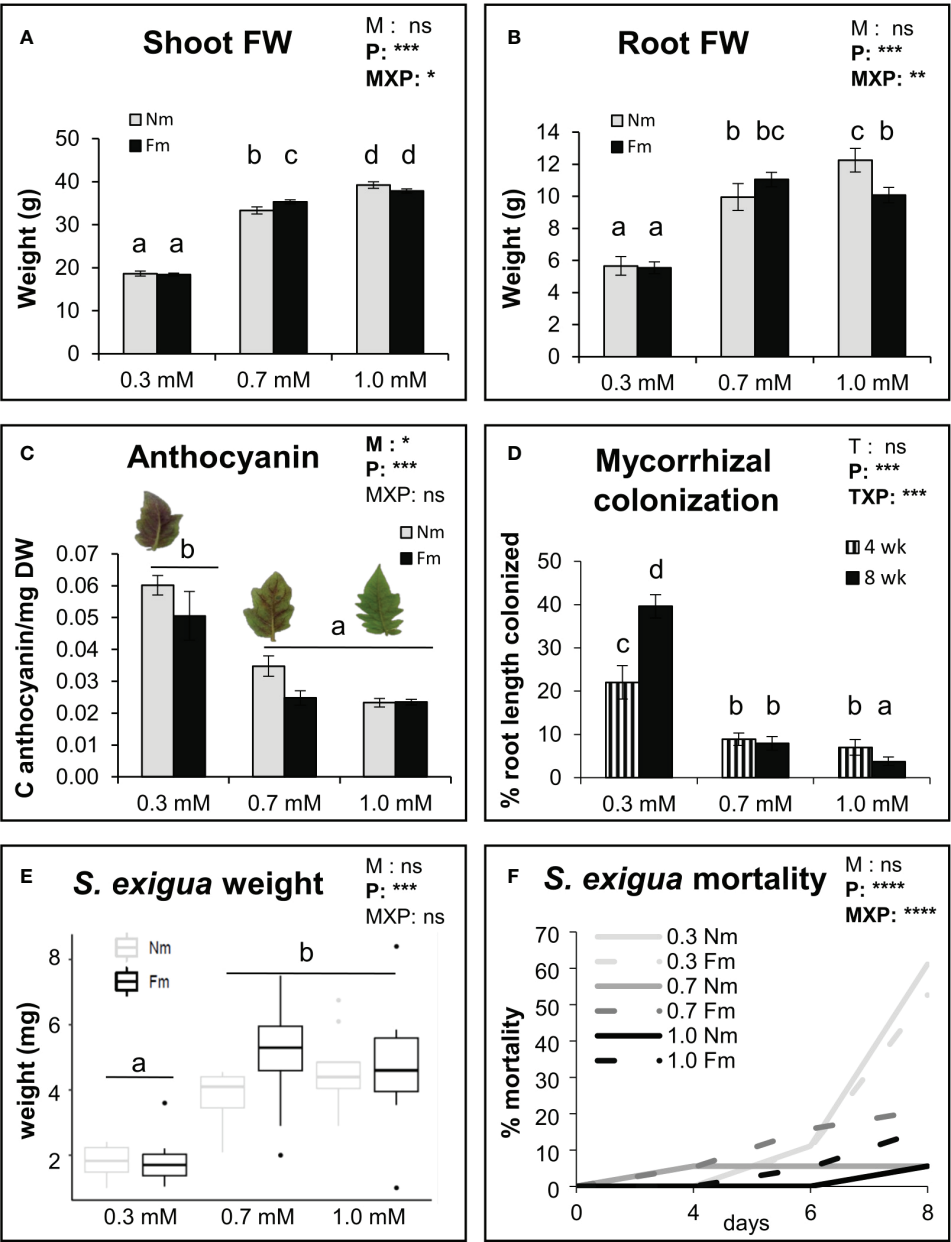
Impact of P levels on plant growth, AM colonization and herbivore performance. Plant growth parameters, fungal colonization and herbivore performance on non-mycorrhizal (Nm) and mycorrhizal tomato plants colonized by *Funneliformis mosseae* (Fm). Plants were fertilized with different P concentrations: 0.3 mM, 0.7 mM and 1mM of H_2_NaPO₄. **(A)** Shoot and **(B)** root fresh weight (n=10), and **(C)** anthocyanin content (n=6) were determined in tomato plants at harvest, 8 weeks post mycorrhizal inoculation (pmi). **(D)** Percentage of root length colonized by the mycorrhizal fungi at 4 and 8 weeks pmi (n=10). One leaf per plant was detached 8 weeks pmi and infested with 2 second instar *S. exigua* larvae (n=20), and **(E)** weight of the larvae was determined after 6 days of feeding. **(F)** Larval mortality was monitored during 8 days of continuous feeding on the detached tomato leaves. Data from A to E represent means of the n independent biological replicates ± SD. Two-way factorial ANOVA **(A–C, E, F)** using AM symbiosis (M) and P treatments (P) as factors, or **(D)** using time (T) and P treatments (P) as factors were performed, and significance values of each factor and their interactions are indicated in the upper right corner of each graph. Asterisks denote significant effect of a factor and their interaction. ns: no significant; *: p < 0.5; **:p < 0.01; ***:p < 0.001; ****:p < 0.0001. Different letters represent statistically significant differences (ANOVA, Fisher’s Least Significant Difference (LSD) test; p<0.05). For (F), data represent the percentage of mortality at the different time points, and the differences in the survival distribution according to P, M and MxP were performed using the non-parametric Log-rank (Mantel-Cox) test.

To explore how mycorrhizal development and its effects on plant and caterpillar performance are affected by P fertilization, we compared 3 fertilization regimes differing only in the P content, ranging from limiting to sufficient P (0.3, 0.7 and 1.0 mM). Analysis of the plant biomass confirmed that P levels had a significant impact on plant growth (*p*<0.001) (**Figures 1A, B**). Plants grown at 0.3 mM P showed about 50% reduced root and shoot weights. Significant differences between 0.7 and 1.0 mM P were also observed; however, these were mild compared to the most P limiting conditions (**Figures 1A, B**). Interestingly, mycorrhization did not have a global significant effect on plant fresh weight, but there was a significant interaction between the P and mycorrhizal treatments, with mycorrhiza promoting plant growth only at the intermediate (0.7 mM) P level and repressing root biomass at the highest P level (Two way ANOVA, **Figures 1A, B**). The evaluation of anthocyanin accumulation in leaves, as an indicator of plant P-starvation response, also confirmed the dose-dependent effects of P fertilization on the plants. Plants grown under low P (0.3 mM) showed the highest anthocyanin levels, while the levels in plants growing at 0.7 and 1.0 mM were not significantly different (**Figure 1C**). Mycorrhization also had a significant effect reducing the anthocyanin levels (two-way ANOVA; *p*<0.05). Mycorrhizal colonization was also significantly impacted by P fertilization, with increasing P concentrations leading to a reduction in colonization (**Figure 1D**). Differences were significant already after 4 weeks of growth, with mycorrhizal colonization in the moderate and high P conditions being half of those at low P. The effect was more pronounced at the later time point (final harvest, 8 weeks), as root colonization continued to increase in plants fertilized with the lowest P concentration (0.3 mM), but not with the other P levels (**Figure 1D**).

The effect of plant P fertilization on the performance of *S. exigua* larvae fed on leaves of those plants was also evaluated by using detached leaves. Larval weight was influenced by P levels, being significantly lower at 0.3 mM P (**Figure 1E**). No differences between larvae fed in 0.7 or 1.0 mM were found, regardless of the mycorrhizal status of the plant (**Figure 1E**). P levels also had a significant impact on *S. exigua* mortality, showing the highest mortality at the low 0.3 P level (ranging between 50-60%) (**Figure 1F**). At higher P levels (0.7 and 1.0 mM) larvae mortality ranged from 6 to 21% (**Figure 1F**). Although higher mortality was found in Fm compared to Nm plants at these medium and high P levels (21% Fm vs 6% Nm for 0.7mM, and 16%Fm vs 6%Nm for 1.0 mM), the differences were not significant. Noteworthy, when analyzing the data separately according to the mycorrhizal status, P effect on larval survival was more pronounced in Nm than in Fm plants: P levels had a very significant impact on larval survival when feeding on Nm plants (*p*<0.0001), but not on those feeding in mycorrhizal plants (*p*=0.052) (**Figure S1A**).

### P effects on herbivore performance depends on the mycorrhizal status of the plant and determine MIR

**Figure 2:**
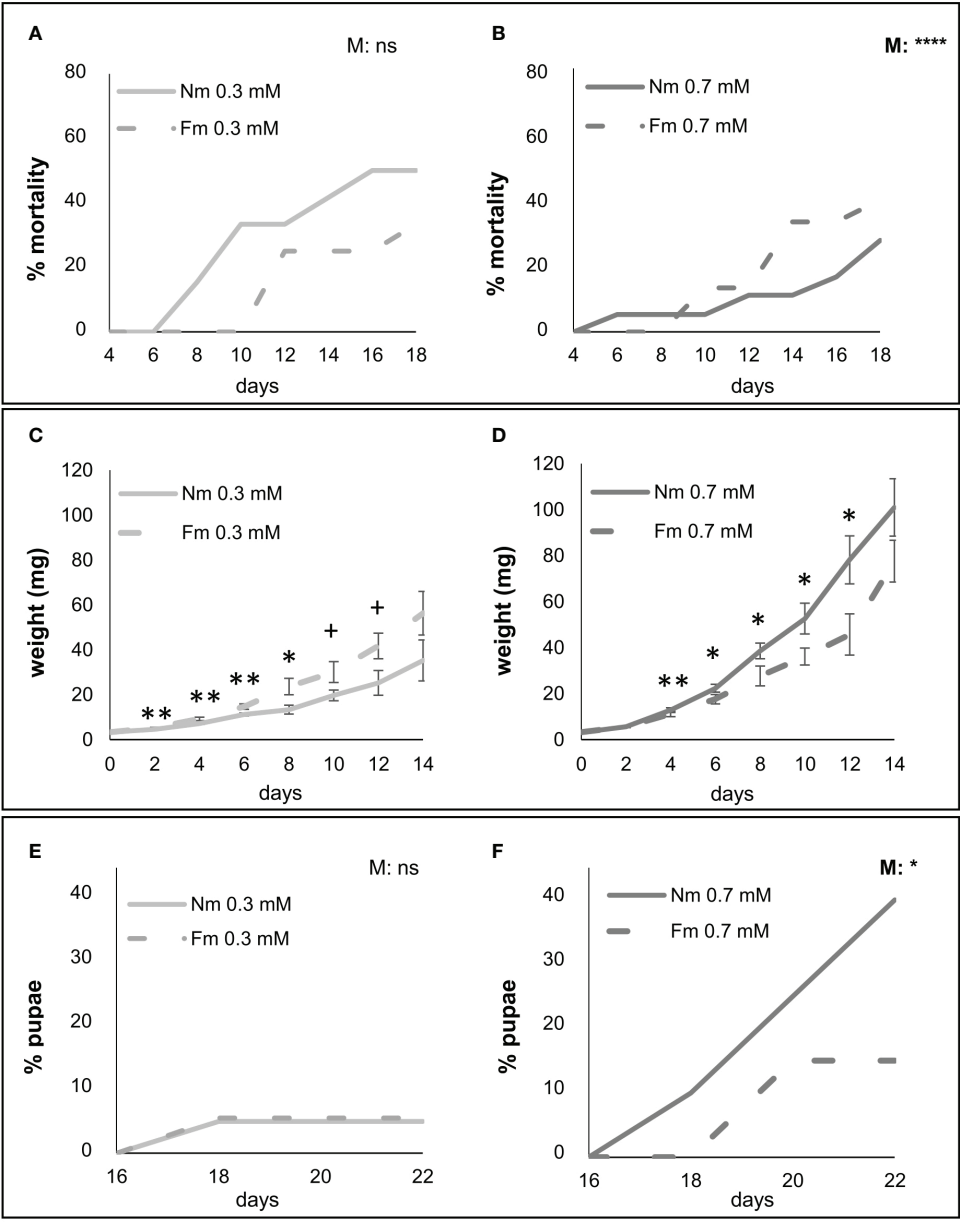
Impact of mycorrhiza on herbivore performance under different P availability. *S. exigua* performance of larvae fed on leaves of mycorrhizal (Fm, dotted line) and non-mycorrhizal (Nm, continuous line) plants fertilized with 0.3 or 0.7 mM of P, light and dark grey, respectively; (n=9). Six-weeks post-inoculation with *F. mosseae* (Fm), plants were infested with second instar *S. exigua* larvae (two per plant, n=18 per treatment) using a clip-cage to confine the larvae to a leaflet. Infestation was maintained for three weeks by moving the clip-cage every two days. **(A, B)** *S. exigua* mortality **(C, D)** weight and **(E, F)** individuals reaching pupa stage. Statistical analyses were performed independently for each P fertilization level: **(A, E)** 0.3 mM and **(B, F)** 0.7 mM. **(A– F)** P values in the upper right corner of each graph indicate statistically differences in mortality and pupation between Nm and Fm, according to Log-rank (Mantel-Cox) test. For larval biomass **(C, D),** values are the weight mean of survived larvae ± SD. Asterisks indicate significant differences between Nm and Fm treatments at given time point according to t-test. ns: no significant; +:p < 0.1; *:p < 0.05; **:p < 0.01; ***:p < 0.001; ****:p<0.0001.

**Figure 3:**
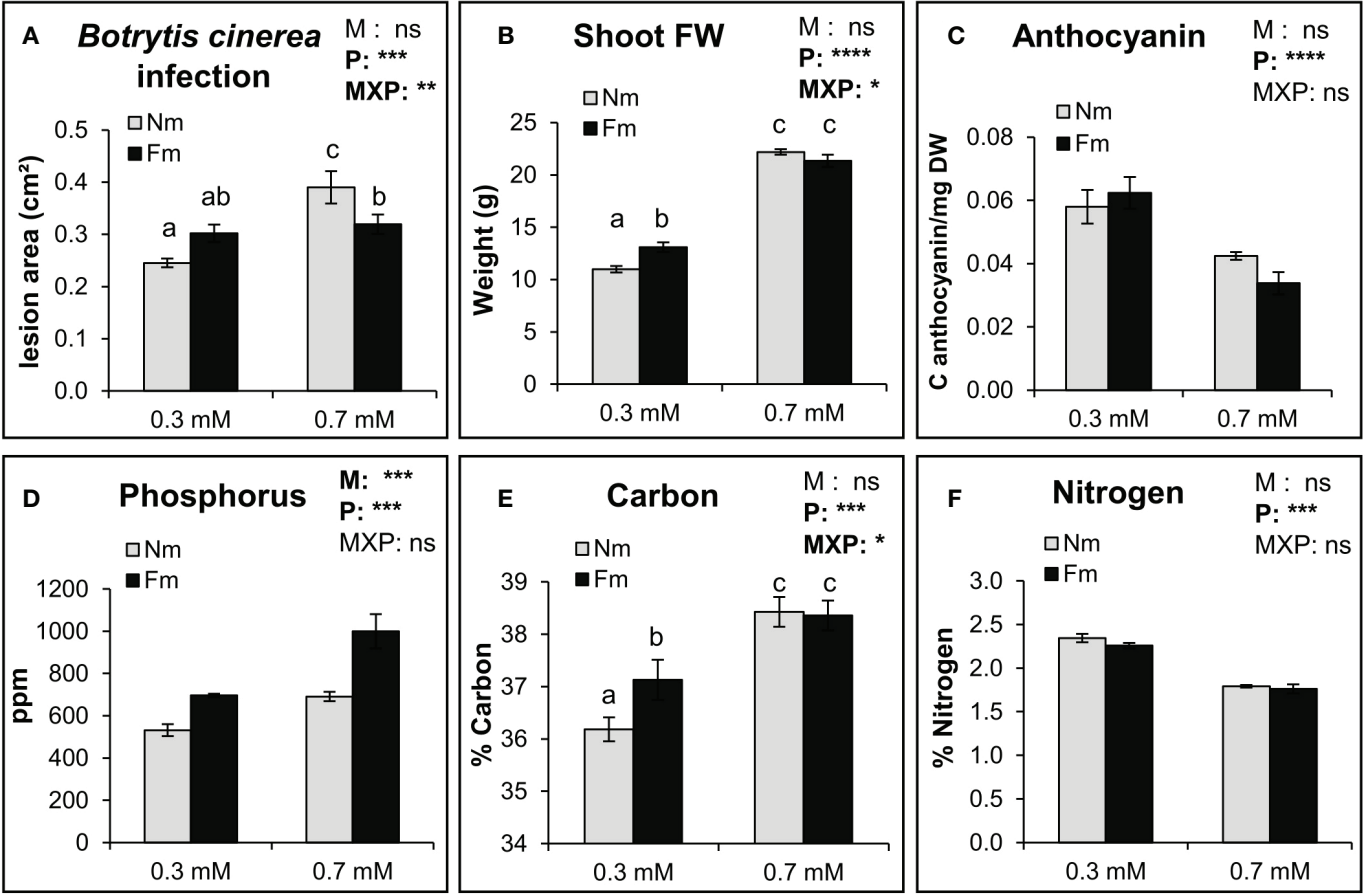
Impact of P fertilization and mycorrhization on the nutritional value of tomato leaves. *Botrytis cinerea* infection, shoot biomass, anthocyanin, and nutrient contents in tomato leaves of non-mycorrhizal (Nm) and mycorrhizal tomato plants colonized by (*F*) *mosseae* (Fm). Plants were fertilized by two P concentrations: 0.3 mM and 0.7 mM P. **(A)** Diameter of necrotic lesions 3 days post inoculation with **(B)** *cinerea* in detached adult leaves from tomato (n=9). **(B)** Shoot fresh weight (n=9), **(C)** anthocyanin (n=6), **(D)** phosphorus (n=6), **(E)** carbon (n=6) and **(F)** nitrogen (n=6) content were measured in tomato plants 6 weeks post mycorrhizal inoculation. Data represent the means of n independent biological replicates ± SD. Two-way ANOVA with mycorrhizal (M) and P treatments (P) as factors, was performed, and the significance of the factors and their interaction is indicated in the upper right corner of each graph. Different letters represent statistically significant differences (ANOVA, Fisher’s Least Significant Difference (LSD) test; *p*<0.05) where the interaction between factors was observed. Otherwise, asterisks denote significant effect of a factor and their interaction. ns: no significant; *: p<0.05; **: p<0.01; ***: p<0.001; ****: p<0.0001.

Overall, the results of the P dose screening revealed that most differences occur between the low (0.3mM) and the higher P regimes (0.7 and 1.0 mM) that showed similar values for most parameters. Accordingly, 0.3 and 0.7 mM levels were selected for further experiments as the closest doses with contrasting effects. Bioassays on detached leaves are useful for quick screenings of major effects, but these effects are usually weaker than those using whole plant bioassays. The latter allow a more realistic set up and longer evaluation periods. Therefore, we performed a second experiment focused on the selected P levels (0.3 and 0.7 mM) to better address the effect of mycorrhization on larval performance under different P fertilization. Mycorrhizal colonization by *F. mosseae* was 14% and 6% for the 0.3 and 0.7 mM P levels, respectively. Again, P effect on *S. exigua* mortality was significant for larvae feeding in Nm plants (*p*<0.0001), but not for those feeding on mycorrhizal (Fm) ones (*p*=0.27) (**Figure S1B**). Thus, mycorrhizal colonization seems to buffer the strong effect of P on plant resistance to the pest. As in the previous experiment using detached leaves, larvae performed worst in the lowest P fertilized plants, showing higher mortality levels (**Figures 2A, B**), lower weight (**Figures 2C, D**) and worst development –evaluated as the percentage of individuals reaching the pupal stage- (**Figures 2E, F**). Regarding the effect of mycorrhization on *S. exigua* performance, under low P no significant changes were found in mortality nor development between Fm and Nm plants (**Figures 2A, E**), and larval weight was even higher at some time points in mycorrhizal plants (**Figure 2C**). In contrast, under moderate P levels (0.7 mM), larvae fed on mycorrhizal Fm plants performed worse than those fed on Nm, showing higher mortality, lower weight and impaired development (**Figures 2B, D, F**). The results reveal that the effect of mycorrhization on larval performance depends on P availability, as MIR was observed at the moderate (0.7 mM) P levels, but not at the low (0.3 mM) one. Remarkably, a similar pattern was observed in the interaction with *B. cinerea*. Again, MIR was only observed at 0.7 mM P, but not at 0.3mM P, and while the effect of P was significant in Nm plants (Nm 0.3 vs Nm 0.7 t-test, *p*<0.0001), it was not significant in Fm plants (Fm 0.3 vs Fm 0.7 t-test, *p*= 0.24) (**Figure 3A**).

### Differences in MIR are not directly related to changes in the nutritional status of the plant

To address whether the P-dependent effects of mycorrhiza on larval performance were due to nutritional aspects, we evaluated plant biomass, anthocyanin and nutrient contents. Shoot biomass was dependent on P fertilization, and growth promotion by mycorrhiza depended on P levels: Fm promoted plant growth at low P, while no plant growth promotion was observed at 0.7 mM P (**Figure 3B**). Anthocyanins were only influenced by P levels (**Figure 3C**). Regarding the nutritional value of the leaves, P content in leaves increased with P fertilization, and it was significantly higher in mycorrhizal plants (**Figure 3D**). P fertilization also significantly influenced carbon (C) and nitrogen (N) levels in leaves, increasing C and reducing N concentration at 0.7mM as compared to 0.3 mM. While mycorrhization did not have a global effect on their content, the interaction between P and M was significant for C content, with mycorrhiza displaying higher C levels than Nm plants under the low P fertilization (**Figures 3E, F**), while the interaction between P and M was significant in C content. As mycorrhization had the same effect on the nutrient content of leaves at the highest P level (higher P values, no changes in C nor N as compared to Nm plants), mycorrhiza-related changes in the nutritional value of leaves do not seem to explain the differential impact of mycorrhization on larval performance under the different P fertilization levels.

Most micronutrients were significantly influenced by P (Ca, Cr, Cu, Fe, Mg, Mn, Mo, Na, Ni, S, Sr and Zn), while M influenced only some of them (as Cu, Fe, K, Li, Mn, Sr, Zn) (**Table S3**). Only K was significantly regulated by the interaction of the two factors (**Table S3**).

### P availability impacts the levels of defense-related phytohormones and gene expression

**Figure 4:**
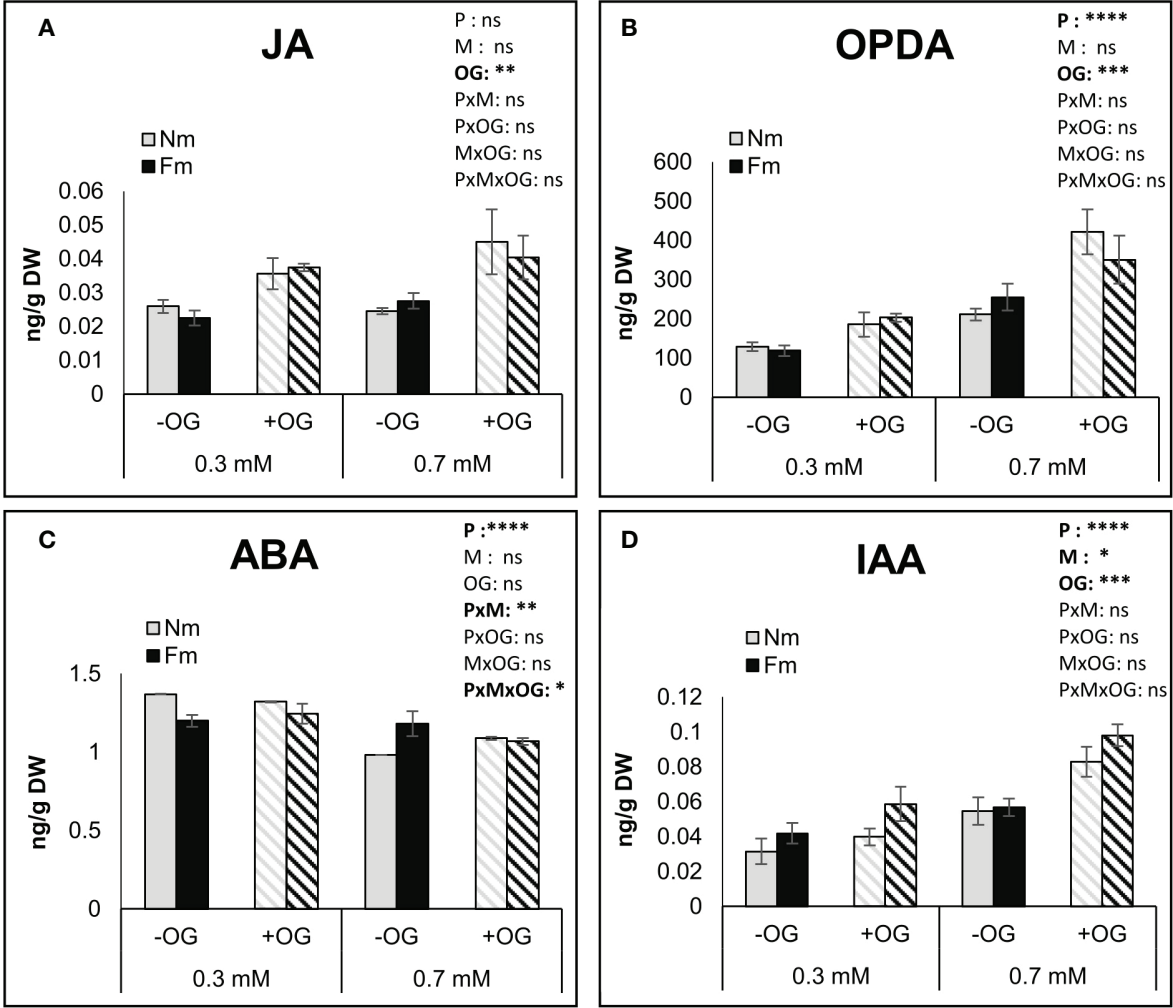
Impact of OG treatment on the content of defense-related phytohormones in tomato leaves. Targeted analysis of phytohormones in non-mycorrhizal (Nm) and mycorrhizal tomato plants colonized by (*F*) *mosseae* (Fm) grown under 0.3 mM or 0.7 mM P fertilization. The fourth true leaf of each plant was sprayed with 50 μg/ml OG solution (+OG; stripped bars) or water for the control (-OG; filled bars) and harvested at 6h post elicitation. **(A)** JA, **(B)** OPDA, **(C)** ABA and **(D)** IAA were quantified by UPLC-MS/MS in tomato leaves. Data represent the means of 6 independent biological replicates ± SD. Three-way ANOVA using P fertilization (P) mycorrhizal (M) and OG treatments (OG) as factors was performed and significance is indicated in the upper right corner of each graph. Asterisks denote significant effect of a factor or their interaction. ns: no significant; *:p < 0.05; **:p < 0.01; ***:p < 0.001; ****:p < 0.0001.

We explored whether the impact of P levels on MIR was related to differential activation of plant defense responses. In order to monitor early defense responses, and aiming to reduce the variability associated with pathogen development or differential feeding by the larvae, we used OGs as elicitors. Plants grown in parallel to those in the whole plant herbivory assay (thus same age, growing conditions and mycorrhizal colonization levels) were challenged by spraying a fully expanded leaf with an OG solution, as described in Gamir et al. (2020). Treated leaves were analyzed for hormone content and defense-related gene expression 6 hours after OG application. The levels of jasmonic acid (JA), its precursor OPDA, auxin (IAA) and abscisic acid (ABA), as the major hormones involved in plant responses to chewing herbivores and necrotrophic pathogens, and described to be regulated by OGs, were evaluated (**Figure 4**). Multiway ANOVA confirmed a significant impact of P fertilization on the OPDA, ABA and IAA levels, but not on the JA content. AM symbiosis only impacted the IAA levels, while OG treatment significantly affected all hormone levels except for ABA. Noteworthy, no significant interaction between the different factors was observed for hormones except for ABA levels. Overall, P deficiency significantly reduced OPDA and IAA levels, but increased ABA. OGs enhanced JA, OPDA and IAA levels regardless of the mycorrhizal status of the plant (**Figure 4**). Mycorrhization only had a significant effect on IAA levels, although it modulated the impact of P and OG on ABA levels (**Figure 4**). Salicylic acid (SA) levels were also determined, showing a reduction by increasing P or OG treatment, but no significant effect of mycorrhiza or factors interaction was observed (**Figure S2**).

**Figure 5:**
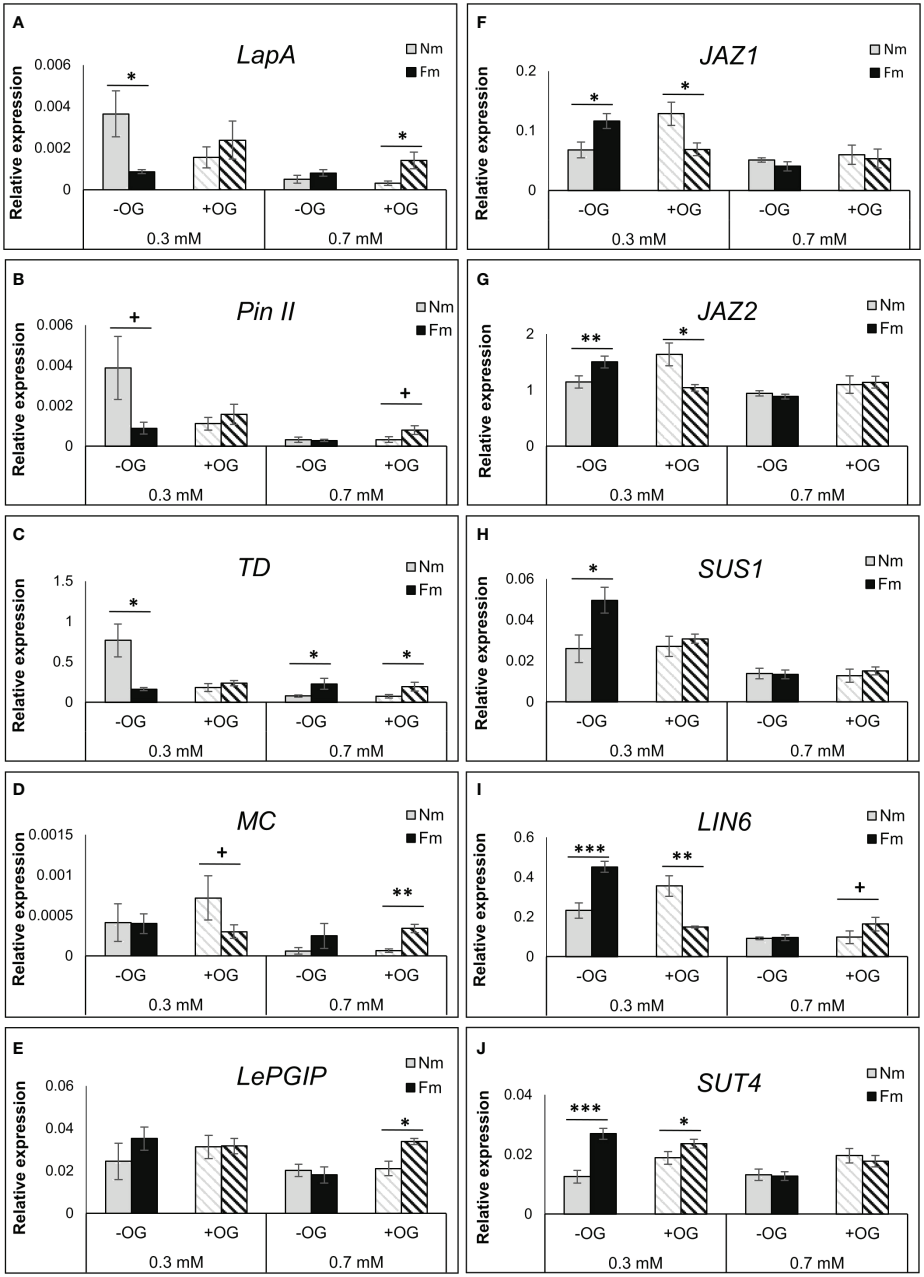
Transcriptional regulation of defense- and growth-related genes. Gene expression levels in OG-treated (+OG; stripped bars) or water-treated (-OG; filled bars) leaves of non-mycorrhizal (Nm) and mycorrhizal (Fm) plants grown under 0.3 mM and 0.7 mM P fertilization regimes. Realtime quantitative RT-qPCR analysis of genes coding for the **(A)** leucyl aminopeptidase A (LapA), **(B)** proteinase inhibitor II (PinII), **(C)** threonine deaminase (TD), **(D)** multicystatin (MC), **(E)** polygalacturonase inhibiting protein (LePGIP), **(F)** JASMONATE ZIM DOMAIN 1 (JAZ1), **(G)** JASMONATE ZIM DOMAIN 2 (JAZ2), **(H)** sucrose synthase-1 (SUS1), **(I)** cell wall invertase 6 (LIN6) and **(J)** sucrose transporter 4 (SUT4) are shown.Values were normalized to the tomato housekeeping gene b-tubulin. Bars represent mean ± SD from 6 biological replicates. Asterisks indicate significant differences between Nm and Fm within the same treatment according to t-test. +: p<0.1; *: p<0.05; **: p<0.01; ***: p<0.001.

As hormone levels were not different in leaves of mycorrhizal plants, we hypothesized that mycorrhizal plants could prime downstream defense responses, but that this effect was dependent on P levels. We analyzed the gene expression of well characterized JA regulated anti-herbivore defense markers, including the *Leucyl aminopeptidase A (LapA*), *Proteinase inhibitor II* (*PinII*), *Threonine deaminase* (*TD*) and *Multicystatin* (*MC*) (Uppalapati et al, 2005); Yan et al, 2013. Surprisingly, in non-challenged plants (-OG) all these defense genes (except MC) were up-regulated in Nm plants grown under low P levels as compared to Nm grown under moderate P levels, but this upregulation was not observed in mycorrhizal Fm plants (**Figures 5A–E**; **Tables S4** and **S5**). OG treatment in plants grown under low P resulted in a reduced expression of these genes in Nm plants, while they showed a slight induction in Fm plants (see fold changes in **Table S5**). In contrast, under moderate (0.7 mM) P levels, these genes showed similar expression levels in mycorrhizal and non-mycorrhizal plants in the absence of challenge; however they were significantly induced by OG treatment only in Fm plants. Thus, the expression analyses confirmed a primed response of mycorrhizal plants to the OG treatment under sufficient P (**Figures 5A–D**; **Table S5**). A similar primed response was found for the gene encoding a defense polygalacturonase inhibiting protein (*LePGIP*), also related to defense responses (Baroncelli et al., 2016) (**Figure 5E**).

The reduced levels of defense genes in non-challenged (-OG) mycorrhizal plants under P starvation, led us to explore the expression of regulators of JA-dependent defense responses. JAZ proteins are key negative regulators of JA signaling, repressing JA-regulated defenses and promoting plant growth (Guo et al., 2018). We checked the genes coding for *JAZ1* and *JAZ2*, both described to be JA-responsive in tomato leaves (Chini et al., 2017). These genes were indeed differentially regulated in mycorrhizal plants under low P, while no differences were found at sufficient P levels (**Figures 5F, G**; **Table S5**). Remarkably, in the absence of challenge (-OG), mycorrhizal plants showed higher expression levels of these negative regulators than Nm plants, in agreement with the repressed expression of the defense genes. However, when plants were elicited with OGs, *JAZ* expression was reduced in mycorrhizal plants, thus releasing the suppression of JA defenses (**Figures 5F, G**; **Table S5**). This effect was illustrated by the increased expression of the JA-regulated defense genes *LapA, PinII* and *TD* in Fm plants when comparing elicited (+OG) with non-elicited (-OG) plants (**Figures 5A–D**, **Table S5**). Since JAZs negatively regulate JA-dependent defenses and modulate the growth-defense balance (Guo et al., 2018), we explored whether the differential *JAZ* expression was associated with transcriptional differences related to primary metabolism. For that, we analyzed genes involved in carbohydrate metabolism and transport: sucrose synthase 1 (*SUS1*), cell wall invertase 6 (*LIN6*) and sucrose transporter 4 (*SUT4*) (Sanmartín et al., 2020). Under low P, they all showed a very strong up-regulation in non-elicited mycorrhizal plants, while the difference was absent (or even reversed as for *LIN6*) upon OG elicitation (**Figures 5H–J**). In contrast, these genes were unaltered by mycorrhizal colonization at high P levels.

The multiway ANOVA of the expression data confirmed the very significant impact of P levels in all genes analyzed, while significant global effects of OG treatment and mycorrhizal status were restricted to specific genes. However, the interaction between mycorrhization and P levels, and between mycorrhization and OG treatment was significant for most genes, confirming that changes in gene expression triggered by both P and OG are modulated by the mycorrhizal status (**Table S6**). Indeed, a strong increase of JA-dependent defenses occurs in Nm plants under P deficiency in the absence of challenge, while they were down-regulated in mycorrhizal Fm plants as compared to Nm plants in these conditions. This defense suppression in Fm plants is likely related to the highest expression of the negative regulators *JAZ1* and *2*, and a transcriptional up-regulation of the carbohydrate metabolism, while no differences were found under sufficient P (**Figure 5**; **Table S5**). In contrast, upon OG treatment, Nm plants did not elicit defenses at any of the P doses (**Table S5**). Plant defenses were only induced in Fm plants, leading to a clear primed defense pattern in mycorrhizal plants under sufficient P fertilization. The transcriptional data suggest a prioritization of the primary metabolism (growth) over defense under P limiting conditions in mycorrhizal plants in the absence of challenge. In contrast, defense responses are prioritized upon challenge, leading to a clear primed response only under sufficient P levels (**Figure 5**; **Tables S4**, **S5**, **S6**).

## Discussion

Evidence of the major role of P in regulating plant immunity is increasing in recent years (Campos-Soriano et al., 2020; Chan et al., 2021; Val-Torregrosa et al., 2022). To identify key molecular regulators and their function, studies usually compare very extreme P levels, addressing null P to dissect P starvation responses (Dixon et al., 2020; Higo et al., 2020). However, even moderate differences in P concentration may have an impact on plant physiology, and severe P deficiencies are unlikely to occur in agricultural settings. In the present study, we assess how moderate differences in P fertilization, which are more likely to occur in agricultural conditions than severe ones, affect mycorrhizal colonization, growth, and resistance to *S. exigua* in tomato, using varying P levels that still allow a good plant development. Our results illustrate that while P regulates plant fitness and pest resistance, its effects depend on the mycorrhizal status of the plant. Moreover, they confirm that plant protection by mycorrhiza depends on P availability, highlighting that P fertilization is a key element in the context-dependency of MIR.

We show the dose-dependent effects of P on tomato growth and resistance against *B. cinerea* and *S. exigua*. Changes in most parameters were not linearly related to the P supply (0.3, 0.7 and 1.0 mM). Instead, P-related differences in most parameters (including plant biomass, anthocyanin content, mycorrhizal colonization, pathogen and herbivore performance on plant) were more pronounced when comparing 0.3 and 0.7 mM P, with no significant differences between 0.7 and 1.0 mM P -except for shoot fresh weight. Therefore, we selected 0.3 and 0.7 mM P as limiting and sufficient P conditions, respectively, to address the mechanisms that may underlie the differences observed in the plant resistance to the herbivore and in MIR under different P scenarios. We observed that the moderate P fertilization (0.7 mM) allowed mycorrhizal colonization while maintaining good plant development.

The inhibitory role of P on mycorrhizal colonization is well documented but varies with the plant and AMF species (Smith and Smith, 2011; Chen et al., 2014; Higo et al., 2020). Mycorrhizal colonization in tomato is indeed highly dependent on P levels. In our study, the medium and high P doses allowed mycorrhizal establishment, but prevented the progression of mycorrhizal colonization in time. However, although mycorrhizal colonization was around 10%, this was enough to significantly affect several parameters. Some growth promotion was observed at the moderate P level, and anthocyanin content, a good indicator of P starvation, were significantly reduced by mycorrhiza under these conditions. The relationship between the extension of mycorrhizal colonization in roots and the benefits of symbiosis has been discussed for years (Treseder and Kathleen, 2013). While higher colonization levels can lead to a stronger P uptake by the mycorrhizal pathway, benefits are not proportional to colonization, and even low colonization levels may have important benefits for the plant.

Regarding the attackers performance, a clear effect of P fertilization was observed. P levels affect both *B. cinerea* lesion development and *S. exigua* performance. Caterpillars performed worst on plants growing under low P, displaying very low weight and very high mortality compared to the other fertilization regimes. This is in agreement with P being an essential nutrient also for caterpillars (Perkins et al., 2004), as previously shown in other caterpillar-plant systems as *Mamestra brassicae*-*Plantago lanceolata* or *Spodoptera littoralis-Nicotiana benthamiana* (Khan et al., 2016; Qu et al., 2021). In our system, mycorrhization *per se* did not affect any of the parameters, but the data analyses revealed a significant interaction between the factors P and mycorrhiza (M). Remarkably, the impact of P levels on larval performance was significant in non-mycorrhizal plants, but not in mycorrhizal plants. A similar pattern was found in the interaction with the pathogen: lesion development was significantly impacted by the P levels in Nm plants, but not in Fm plants. Thus, the mycorrhizal symbiosis buffered the influence of P fertilization on the susceptibility to the attacker. Because of the limitations of performing herbivory tests in detached leaves, we performed more detailed analyses allowing the caterpillars to feed directly in the plant using the low (0.3 mM) and moderate (0.7 mM) P doses, which previously showed contrasting effects on plant and *S. exigua* performance. Using whole plants, not only local, but also systemic plant responses may contribute to the final outcome, as for example roots and distal leaves play a key role in response to damage and to herbivory (Kundu et al., 2018; Erb and Reymond, 2019; Gamir et al., 2020).

Under the lowest P level, there was no effect of the mycorrhizal symbiosis on larval mortality nor on their development to pupae. Noteworthy, caterpillar weight was higher for those feeding on mycorrhizal plants than for those on non-mycorrhizal ones, pointing to a potential benefit of the AM symbiosis at the nutritional level. In contrast, under 0.7 mM P, functional MIR was observed, as the symbiosis with *F. mosseae* reduced *S. exigua* survival, weight and the proportion of individuals reaching the pupa state. The prevalence of MIR at 0.7 but not at 0.3 mM P was also observed in the interaction with *B. cinerea.* Thus, the results show that mycorrhiza effects on plant resistance to pathogens and herbivores performance are P-dependent, and that MIR was impaired under P deficiency. A modulatory role of nitrogen deficiency on MIR against pathogens was also previously shown in tomato (Sanchez-Bel et al., 2016).

The effects of mycorrhization on plant interaction with herbivores are very complex. Enhanced resistance against chewing herbivores had been described in multiple systems (Schoenherr et al., 2019; Selvaraj et al., 2020; Jiang et al, 2021; Shafiei et al, 2022), but opposite effects have also been reported (Bennett et al., 2009; Hoffmann et al, 2009; Bernaola et al., 2018; Zeng et al., 2022), as the improved nutritional status of AM plants may benefit insects (Koricheva et al, 2009; Pozo et al., 2020). To understand the mechanisms underlying the contrasting effects of P availability on AM impact on insect performance, we investigated several plant nutritional and defensive traits that may contribute to the observed resistance phenotypes. Regarding plant nutrition and growth, mycorrhizal colonization enhanced shoot growth at low P doses and significantly elevated P levels in the plants regardless of the fertilization doses. C and N, recognized as key limiting-nutrients for insect herbivores (Mattson, 1980), were both influenced by P, but mycorrhiza did only change C levels under low P fertilization. The increase by mycorrhiza in C content under low P may have contributed to the better performance of the larvae in mycorrhizal plants under the lowest fertilization conditions. However, the contribution of changes in these nutrients to the worst performance of the herbivore in mycorrhizal plants under the 0.7 mM P is unlikely, since P is increased -and this would be positive for the pest- and C and N did not differ between Nm and Fm plants. Nevertheless, other nutritional aspects, such as the changes in other micronutrients, and potential variations in the content of sugars and amino acids upon herbivory may have contributed to the resistance phenotype and deserve further attention in follow-up studies. In fact, nutrition related changes, including protein and amino acid contents in mycorrhizal plants, have been proposed to contribute to resistance against *S. exigua* in maize plants (Ramírez-Serrano et al, 2022). In tomato, significant changes in sugar and amino acid contents occurs in response to herbivory in systemic tomato leaves may contribute to defense (Kundu et al., 2018). Whether this systemic regulation is modulated in mycorrhizal plants remain to be determined.

P effects can go beyond nutritional aspects. P starvation has been associated with an increase in JA signaling and expression of JA-related genes in different plant species, potentially leading to increased defense against pathogens and chewing herbivores in Arabidopsis (Khan et al., 2016; Luo et al., 2021). Although we found lower *B. cinerea* lesion development and higher mortality of *S. exigua* when feeding in plants under deficient (0.3 mM) P as compared to the medium 0.7 mM dose, no basal changes in the JA content among these plants were observed. Only the JA precursor OPDA differed among those plants, with higher levels at the higher P doses. Therefore, increased mortality at low P is not likely to be explained by a higher basal JA accumulation under such growing conditions, but rather by poor quality of the leaves (e.g. low nutrients and high anthocyanins) or by a stronger activation of defenses in those leaves as indicated by the transcriptional analysis of marker genes discussed below. The levels of IAA and ABA were also evaluated, as they are also regulated during responses to herbivory (Vos et al., 2013; Machado et al., 2016; Erb and Reymond, 2019). P fertilization impacted ABA, IAA and SA levels in the leaves, with ABA and SA concentrations being higher and IAA lower under low P conditions. Mycorrhizal colonization did not alter hormone basal levels except for a slight increase in auxins at P deficiency. Accordingly, basal differences in phytohormone levels among mycorrhizal and non-mycorrhizal plants are not likely to mediate the observed mycorrhizal effect on pathogen and larval performance under moderate P conditions.

We then addressed whether the differences may be related to a differential plant capacity to activate defenses. In order to analyze early defense responses and avoid variability associated with pathogen and caterpillar preference, we chemically elicited damage responses by application of OGs. OGs are released from the plant cell wall by pathogen polygalacturonases (Cervone et al., 1989; Ferrari et al., 2013), but also by endogenous polygalacturonases induced upon wounding, as shown in multiple plant species including tomato (Bergey et al., 1999; Orozco-Cárdenas and Ryan, 1999; De Lorenzo et al., 2011). As chewing herbivores elicit wound responses in the plant, they are also thought to trigger OGs release (Aljbory and Chen, 2018; Erb and Reymond, 2019) although this assumption needs further experimental evidences. Endogenous and exogenously applied OG elicitors activate plant defense responses and enhance resistance to different aggressors (De Lorenzo et al., 2011; Benedetti et al., 2015; Gamir et al., 2020). In the present study, OG application promoted JA, OPDA and IAA accumulation in leaves from plants under both, low and moderate, fertilization levels. Furthermore, OPDA and IAA levels were higher under the higher P fertilization, while JA was not affected by P availability. Although JA, OPDA and IAA increased their content under elicitation, no differences in their levels between Fm and Nm plants were found. We further analyzed well characterized JA regulated, anti-herbivory defense marker genes as those coding for *LapA, Pin II, MC* and *TD* (Uppalapati et al, 2005; Yan et al, 2013). All these marker genes showed a priming profile in mycorrhizal plants under 0.7 mM P, with a significantly higher expression upon OG treatment than in Nm plants. Conversely, under the most limiting P level, most of those genes showed much higher expression in non-elicited Nm plants, suggesting that Nm plants activate JA-related defenses upon P starvation in the absence of damage elicitation. It should be noted that although no differences were found between mycorrhizal and non-mycorrhizal plants elicited with OGs under the lowest P level (0.3 mM), there were differences when considering the fold change between non-elicited and elicited plants within each type of plants. While Nm plants showed a repression of defense-related genes upon OG treatment under P-deficient conditions, mycorrhizal Fm plants showed an increase in their expression. Thus, the main difference is the basal, non-elicited status of these plants: Nm plants are expressing JA-dependent defenses at the basal level, but fail to elicit defenses upon elicitation, in contrast to the behavior of mycorrhizal plants. The enhanced growth promotion of mycorrhizal plants under the lowest P fertilization, higher P content in leaves and lower anthocyanin levels suggest that mycorrhizal plants prioritize the improvement in nutrition under P deficiency, but prioritize defense activation under non-deficient conditions. Priming of plant defenses against herbivores has been shown in different systems (Song et al, 2013; Rivero et al, 2021; Jiang et al., 2021), but a failure of defense priming has been also reported (Qu et al., 2021; Ramírez-Serrano et al, 2022; Zeng et al., 2022). Analysis of chemical defenses, as the accumulation of polyphenols, lignins and other defensive metabolites in future studies will contribute to better characterize the differences in the defense responses under the different nutrition scenarios. Moreover, the potential contribution of nutrition to the different outcomes in those experiments is difficult to address, as the experimental systems were very different, but it is tempting to speculate that nutrient availability is a key player in defining the final resistance phenotype. The results of the present study support this hypothesis by evidencing contrasting defense-responses in mycorrhizal and non-mycorrhizal plants to elicitation depending on the P nutritional status.

It is well established that when subjected to multiple stresses, plants prioritize their responses to promote growth or defense to optimize their resources according to the specific needs in a given context (Smakowska et al., 2016). JA signaling has been described to be a key player in such prioritization, with negative regulators of JA signaling acting when defenses are not needed (Major et al., 2017; Guo et al., 2018). We explored the regulation of two JAZ transcriptional repressors characterized as JA-inducible in tomato leaves (Chini et al., 2017). We found that while no significant differences related to the mycorrhizal status of the plant were observed at the higher P level, *JAZ* expression was higher in mycorrhizal plants under P limitation, in agreement with the lower expression levels of the defense-related genes *LapA*, *PinII*, *MC* and *TD*. In contrast, upon OG elicitation the expression of *JAZ* genes was suppressed in mycorrhizal plants, likely releasing the repression of the JA signaling pathway to activate plant defenses. Finally, to test whether this role in mediating the defense-growth trade-off reported for JAZ may be leading to differences in the regulation of the primary and secondary metabolism in mycorrhizal plants, we analyzed the expression of carbohydrate-related genes. The genes encoding the sucrose synthase *SUS1*, the invertase *LIN6* and the sucrose transporter *SUT4* (Sanmartin et al, 2020) were all showing higher expression in mycorrhizal plants under P deficiency in the absence of challenge, matching the expression profile found for the *JAZ* genes. These higher expression levels were not maintained upon OG elicitation. Remarkably, no differences were found in these carbohydrate-related genes at moderate P levels. Therefore, mycorrhizal plants showed reduced expression of defense-related genes in the absence of challenge under P-limiting conditions, which correlated with higher expression levels of the genes coding for the JA negative regulators *JAZ1* and *JAZ2* and of genes related to C metabolism. Thus, under P deficiency, mycorrhizal symbiosis drives the plant response towards the activation of primary metabolism, that is, growth-related responses but this effect disappears upon a challenge or pest attack. On the contrary, when P levels are higher, no differences between mycorrhizal and non-mycorrhizal plants are found in the absence of challenge. However, upon damage signaling elicitation defense responses are primed in mycorrhizal plants, thus promoting defenses. Differential regulation of the growth-defense balance in mycorrhizal plants was also shown under N depleted environments (Sanchez-Bel et al., 2018). Thus, experimental evidences support a fine-tuned regulation of the growth-defense balance in mycorrhizal plants according to the nutrients availability.

In summary, our results show that P availability plays a regulatory role in JA-dependent plant defense responses, and the effect is modulated by the mycorrhizal status of the plant. We also show that P availability can be a key element in the reported context dependency of MIR, as mycorrhization effects ranged from moderately positive (increase in larval biomass under the lowest P level) to negative effects on disease and herbivore development (enhanced mortality and reduced biomass and individuals reaching pupa stage) under moderate P levels, thus displaying MIR. Our hormonal and transcriptional analyses point to a differential regulation of the growth-defense balance in mycorrhizal plants, where the symbiosis tailors the plant to prioritize primary metabolism and nutritional-growth related responses under P deficiency, but promotes priming of plant defenses when this key element is not limiting. This fine-tuned regulation of defenses may contribute to the enhanced resilience of mycorrhizal plants under varying conditions and multi-stress contexts. Understanding the underlying molecular mechanisms will contribute to improve the efficacy of mycorrhizal inoculants in crop protection.

## Data availability statement

The original contributions presented in the study are included in the article/**Supplementary Material**. Further inquiries can be directed to the corresponding author.

## Author contributions

LD, JAL-R, and MP conceptualized the study. LD performed plant and insect bioassays, physiological, biochemical, and gene expression analyses. JR collaborated in the performance of the experiments and JG was responsible of the hormone analyses. LD and BR-S performed statistical analyses. All authors contributed to data interpretation and paper redaction. LD and MP wrote the manuscript. All authors contributed to the final version of the article and approved the submission.

## Supplementary material

Supplementary Figure 1 Survival probability curves of *S. exigua* fed on leaves of mycorrhizal (Fm, dotted line) and non-mycorrhizal (Nm, continuous line) tomato plants. **(A)** Larvae were feeding on detached leaves from plants fertilized with 0.3 (light grey), 0.7 (grey) or 1.0 (black) mM of H_2_NaPO₄, one leaf of each plant (n=10) was detached 8 weeks pmi and infested with two second instar *S. exigua* larvae (n=20 larvae). **(B)** Larvae feeding on leaves of plants (n=9) fertilized with 0.3 mM (light grey) or 0.7 mM (grey) of H_2_NaPO₄. Two second instar larvae were added to one leaf of intact plants using clip-cages (n=18 per treatment) 6 weeks pmi, and infestation was maintained for three weeks more. Survival distribution comparisons were performed using the non-parametric Log-rank (Mantel-Cox) test.

Supplementary Figure 2 Salicylic acid content in non-mycorrhizal (Nm) and mycorrhizal tomato plants colonized by *F. mosseae* (Fm) grown under 0.3 mM or 0.7 mM P fertilization, and treated with 50 μg/ml OG solution (+OG; stripped bars) or water as control (-OG; filled bars), harvested 6h post elicitation. Multiway ANOVA using P fertilization (P) mycorrhizal (M) and OG treatment (OG) as factors was performed and significance is indicated in the upper right corner of the graph. Asterisks denote significant effect of a factor or their interaction. ns: no significant; *: p<0.05; **: p<0.01; ***: p<0.001; ****: p<0.0001.

Supplementary Table 1 Nutrient solution used for the experiments, modified from Hewitt (1996) in the total amount of H₂NaPO₄ to obtain the low P (0.3 mM), medium P (0.7 mM) and high P (1.0 mM) P fertilization regimes.

Supplementary Table 2 List of primers used for qPCR analysis.

Supplementary Table 3 Microelement concentration in leaf tissues. Data represent mean values (n=3). For each element (columns), treatments sharing a letter in common are not significantly different according to one way ANOVA followed by Fisher’s Least Significant Difference (LSD) test (p<0.05). The global effects of the factors: AM symbiosis (M), P fertilization (P) and their interaction were analyzed by multiway ANOVA. Asterisks indicate significant differences: *: p<0.05; **: p<0.01; ***: p<0.001; p<0.0001.

Supplementary Table 4 Gene expression data from real-time quantitative qRT-PCR normalized to housekeeping gene ß-tubulin. Gene expression levels in OG-treated or water-treated leaves of non-mycorrhizal (Nm) and mycorrhizal (Fm) plants grown under 0.3 mM and 0.7 mM P fertilization regimes. Values are the mean of 6 biological replicates.

Supplementary Table 5 Fold changes in gene expression. Colors indicate up-regulation (red) and down-regulation (blue) and intensities are determined by the intensity of the changes: light color >1.5 or <0.75 and dark color >2 or <0.5 and significant effects are highlighted in bold. **(A)** Effect of P starvation on gene expression in Nm plants (fold change in Nm and Fm plants fertilized at 0.3 vs 0.7 mM). **(B)** Effect of OG elicitation on gene expression in Fm or Nm plants grown at 0.3 mM and 0.7 mM P (fold change +OG/-OG). **(C)** Effect of AM symbiosis on gene expression at 0.3 mM and 0.7 mM P (fold change Fm/Nm). Bold numbers and asterisks indicate significant differences (t-test, *p*<0.05) between **(A)** 0.3 vs 0.7 mM, **(B)** +OG vs -OG and **(C)** Fm vs Nm. +: p<0.1; *: p<0.05; **: p<0.01; ***: p<0.001.

Supplementary Table 6 Multiway ANOVA of gene expression data. Effects of the different factors: AM symbiosis (M), P fertilization (P) and elicitation (OG treatment, OG) and their interactions were analyzed. Data represent f values and significant effects are highlighted in bold. Asterisks indicate significant differences: +: p<0.1; *: p<0.05; **: p<0.01; ***: p<0.001.

## References

[B1] Aljbory Z. Chen M. S. (2018). Indirect plant defense against insect herbivores: A review. Insect Sci. 25, 2–23. doi: 10.1111/1744-7917.12436 28035791

[B2] Andersen C. L. Jensen J. L. Ørntoft T. F. (2004). Normalization of real-time quantitative reverse transcription-PCR data: A model-based variance estimation approach to identify genes suited for normalization, applied to bladder and colon cancer data sets. Cancer Res. 64, 5245–5250. doi: 10.1158/0008-5472.CAN-04-0496 15289330

[B3] Baroncelli R. Matarese F. Moncini L. Vannacci G. Vergara M. (2016). Two endopolygalacturonase genes in *Trichoderma virens*: In silico characterization and expression during interaction with plants. J. Phytopathol. 164, 18–28. doi: 10.1111/jph.12414

[B4] Benedetti M. Mattei B. Pontiggia D. Salvi G. Savatin D. V. Ferrari S. (2017). Methods of isolation and characterization of oligogalacturonide elicitors. Methods Mol. Biol. 157825–, 38. doi: 10.1007/978-1-4939-6859-6_3 28220413

[B5] Benedetti M. Pontiggia D. Raggi S. Cheng Z. Scaloni F. Ferrari S.. (2015). Plant immunity triggered by engineered *in vivo* release of oligogalacturonides, damage-associated molecular patterns. Proc. Natl. Acad. Sci. 112, 5533–5538. doi: 10.1073/pnas.1504154112 25870275 PMC4418913

[B6] Bennett A. E. Bever J. D. Deane Bowers M. (2009). ). arbuscular mycorrhizal fungal species suppress inducible plant responses and alter defensive strategies following herbivory. Oecologia 160, 771–779. doi: 10.1007/s00442-009-1338-5 19408016

[B7] Berendsen R. L. Pieterse C. M. Bakker P. A. (2012). The rhizosphere microbiome and plant health. Trends Plant Sci. 17, 478–486. doi: 10.1016/j.tplants.2012.04.001 22564542

[B8] Bergey D. R. Orozco-Cardenas M. De Moura D. S. Ryan C. A. (1999). A wound-and systemin-inducible polygalacturonase in tomato leaves. Proc. Natl. Acad. Sci. 96, 1756–1760. doi: 10.1073/pnas.96.4.1756 9990097 PMC15585

[B9] Bernaola L. Cosme M. Schneider R. W. Stout M. (2018). Belowground inoculation with arbuscular mycorrhizal fungi increases local and systemic susceptibility of rice plants to different pest organisms. Front. Plant Sci. 9. doi: 10.3389/fpls.2018.00747 PMC5996305 29922319

[B10] Breuillin F. Schramm J. Hajirezaei M. Ahkami A. Favre P. Druege U.. (2010). Phosphate systemically inhibits development of arbuscular mycorrhiza in *Petunia hybrida* and represses genes involved in mycorrhizal functioning. Plant J. 64, 1002–1017. doi: 10.1111/j.1365-313X.2010.04385.x 21143680

[B11] Campo S. Martín-Cardoso H. Olivé M. Pla E. Catala-Forner M. Martínez-Eixarch M.. (2020). Effect of root colonization by arbuscular mycorrhizal fungi on growth, productivity and blast resistance in rice. Rice 13, 1–14. doi: 10.1186/s12284-020-00402-7 32572623 PMC7310045

[B12] Campos-Soriano L. Bundó M. Bach-Pages M. Chiang S. F. Chiou T. J. San Segundo B. (2020). Phosphate excess increases susceptibility to pathogen infection in rice. Mol. Plant Pathol. 21, 555–570. doi: 10.1111/mpp.12916 32072745 PMC7060143

[B13] Campos-Soriano L. García-Martínez J. San Segundo B. (2012). The arbuscular mycorrhizal symbiosis promotes the systemic induction of regulatory defence-related genes in rice leaves and confers resistance to pathogen infection. Mol. Plant Pathol. 13, 579–592. doi: 10.1111/j.1364-3703.2011.00773.x 22212404 PMC6638712

[B14] Castrillo G. Teixeira P. J. P. L. Paredes S. H. Law T. F. De Lorenzo L. Feltcher M. E.. (2017). Root microbiota drive direct integration of phosphate stress and immunity. Nature 543, 513–518. doi: 10.1038/nature21417 28297714 PMC5364063

[B15] Cervone F. Hahn M. G. De Lorenzo G. Darvill A. Albersheim P. (1989). Host-pathogen interactions: XXXIII. a plant protein converts a fungal pathogenesis factor into an elicitor of plant defense responses. Plant Physiol. 90, 542–548. doi: 10.1104/pp.90.2.542 16666805 PMC1061758

[B16] Chan C. Liao Y. Y. Chiou T. J. (2021). The impact of phosphorus on plant immunity. Plant Cell Physiol. 62, 582–589. doi: 10.1093/pcp/pcaa168 33399863

[B17] Chen M. Arato M. Borghi L. Nouri E. Reinhardt D. (2018). Beneficial services of arbuscular mycorrhizal fungi–from ecology to application. Front. Plant Sci. 9. doi: 10.3389/fpls.2018.01270 PMC6132195 30233616

[B18] Chen P. J. Senthilkumar R. Jane W. N. He Y. Tian Z. Yeh K. W. (2014). Transplastomic *Nicotiana benthamiana* plants expressing multiple defence genes encoding protease inhibitors and chitinase display broad-spectrum resistance against insects, pathogens and abiotic stresses. Plant Biotechnol. J. 12, 503–515. doi: 10.1111/pbi.12157 24479648

[B19] Chini A. Ben-Romdhane W. Hassairi A. Aboul-Soud M. A. (2017). Identification of TIFY/JAZ family genes in *Solanum lycopersicum* and their regulation in response to abiotic stresses. PloS One 12, e0177381. doi: 10.1371/journal.pone.0177381 28570564 PMC5453414

[B20] Cordell D. White S. (2015). Tracking phosphorus security: indicators of phosphorus vulnerability in the global food system. Food Secur. 7, 337–350. doi: 10.1007/s12571-015-0442-0

[B21] De Lorenzo G. Brutus A. Savatin D. V. Sicilia F. Cervone F. (2011). Engineering plant resistance by constructing chimeric receptors that recognize damage-associated molecular patterns (DAMPs). FEBS Lett. 585, 1521–1528. doi: 10.1016/j.febslet.2011.04.043 21536040

[B22] Diagne N. Ngom M. Djighaly P. I. Fall D. Hocher V. Svistoonoff S. (2020). Roles of arbuscular mycorrhizal fungi on plant growth and performance: Importance in biotic and abiotic stressed regulation. Diversity 12, 370. doi: 10.3390/d12100370

[B23] Dixon M. Simonne E. Obreza T. Liu G. (2020). Crop response to low phosphorus bioavailability with a focus on tomato. Agronomy 10, 617. doi: 10.3390/agronomy10050617

[B24] Dowarah B. Gill S. S. Agarwala N. (2022). Arbuscular mycorrhizal fungi in conferring tolerance to biotic stresses in plants. J. Plant Growth Regul. 41, 1429–1444. doi: 10.1007/s00344-021-10392-5

[B25] Erb M. Reymond P. (2019). Molecular interactions between plants and insect herbivores. Annu. Rev. Plant Biol. 70, 527–557. doi: 10.1146/annurev-arplant-050718-095910 30786233

[B26] Ferrari S. Savatin D. V. Sicilia F. Gramegna G. Cervone F. (2013). And Lorenzo, G Oligogalacturonides: plant damage-associated molecular patterns and regulators of growth and development. D. Front. Plant Sci. 4. doi: 10.3389/fpls.2013.00049 PMC3595604 23493833

[B27] Ferrol N. Azcón-Aguilar C. Pérez-Tienda J. (2019). Arbuscular mycorrhizas as key players in sustainable plant phosphorus acquisition: An overview on the mechanisms involved. Plant Sci. 280, 441–447. doi: 10.1016/j.plantsci.2018.11.011 30824024

[B28] Fiorilli V. Vannini C. Ortolani F. Garcia-Seco D. Chiapello M. Novero M.. (2018). Omics approaches revealed how arbuscular mycorrhizal symbiosis enhances yield and resistance to leaf pathogen in wheat. Sci. Rep. 8, 1–18. doi: 10.1038/s41598-018-27622-8 29941972 PMC6018116

[B29] Frew A. Price J. N. (2019). Mycorrhizal-mediated plant–herbivore interactions in a high CO_2_ world. Funct. Ecol. 33, 1376–1385. doi: 10.1111/1365-2435.13347

[B30] Gamir J. Minchev Z. Berrio E. García J. M. De Lorenzo G. Pozo M. J. (2020). Roots drive oligogalacturonide-induced systemic immunity in tomato. Plant Cell Environ. 44, 275–289. doi: 10.1111/pce.13917 33070347 PMC7883634

[B31] Gamir J. Pastor V. Cerezo M. Flors V. (2012). Identification of indole-3-carboxylic acid as mediator of priming against *Plectosphaerella cucumerina* . Plant Physiol. Biochem. 61, 169–179. doi: 10.1016/j.plaphy.2012.10.004 23116603

[B32] Giovannetti M. Mosse B. (1980). An evaluation of techniques for measuring vesicular arbuscular mycorrhizal infection in roots. New Phytol. 84, 489–500. doi: 10.1111/j.1469-8137.1980.tb04556.x

[B33] Greene G. L. Leppla N. C. Dickerson W. A. (1976). Velvetbean caterpillar: a rearing procedure and artificial medium. J. Econ. Entomol. 69, 487–488. doi: 10.1093/jee/69.4.487

[B34] Gruden K. Lidoy J. Petek M. Podpečan V. Flors V. Papadopoulou K. K.. (2020). Ménage à trois: Unraveling the mechanisms regulating plant–microbe–arthropod interactions. Trends Plant Sci. 25, 1215–1226. doi: 10.1016/j.tplants.2020.07.008 32828689

[B35] Guo Q. Yoshida Y. Major I. T. Wang K. Sugimoto K. Kapali G.. (2018). JAZ repressors of metabolic defense promote growth and reproductive fitness in. Arabidopsis. Proc. Natl. Acad. Sci. 115, E10768–E10777. doi: 10.1073/pnas.1811828115 30348775 PMC6233084

[B36] Ha S. Tran L. S. (2014). Understanding plant responses to phosphorus starvation for improvement of plant tolerance to phosphorus deficiency by biotechnological approaches. Crit. Rev. Biotechnol. 34, 16–30. doi: 10.3109/07388551.2013.783549 23586682

[B37] Hewitt E. J. (1996). “The composition of the nutrient solution,” in Sand and water culture methods used in the study of plant nutrition. technical communication No.22 (London: Commonwealth Agriculture Bureau).

[B38] Higo M. Azuma M. Kamiyoshihara Y. Kanda A. Tatewaki Y. Isobe K. (2020). Impact of phosphorus fertilization on tomato growth and arbuscular mycorrhizal fungal communities. Microorganisms 8, 178. doi: 10.3390/microorganisms8020178 31991824 PMC7074694

[B39] Hoffmann D. Vierheilig H. Riegler P. Schausberger P. (2009). Arbuscular mycorrhizal symbiosis increases host plant acceptance and population growth rates of the two-spotted spider mite *Tetranychus urticae* . Oecologia 158, 663–671. doi: 10.1007/s00442-008-1179-7 18949488

[B40] Jiang D. Tan M. Wu S. Zheng L. Wang Q. Wang Q.. (2021). Defense responses of arbuscular mycorrhizal fungus-colonized poplar seedlings against gypsy moth larvae: a multiomics study. Hortic. Res. 8, 245. doi: 10.1038/s41438-021-00671-3 34848684 PMC8632881

[B41] Jung S. C. Martínez-Medina A. López-Ráez J. A. Pozo M. J. (2012). Mycorrhiza-induced resistance and priming of plant defenses. J. Chem. Ecol. 38, 651–664. doi: 10.1007/s10886-012-0134-6 22623151

[B42] Kaplan E. L. Meier P. (1958). Nonparametric estimation from incomplete observations. J. Am. Stat. Assoc. 53, 457–481. doi: 10.1080/01621459.1958.10501452

[B43] Keymer A. Pimprikar P. Wewer V. Huber C. Brands M. Bucerius S. L.. (2017). Lipid transfer from plants to arbuscular mycorrhiza fungi. elife 6, e29107. doi: 10.7554/eLife.29107 28726631 PMC5559270

[B44] Khan G. A. Vogiatzaki E. Glauser G. Poirier Y. (2016). Phosphate deficiency induces the jasmonate pathway and enhances resistance to insect herbivory. Plant Physiol. 171, 632–644. doi: 10.1104/pp.16.00278 27016448 PMC4854718

[B45] Koricheva J. Gange A. C. Jones T. (2009). Effects of mycorrhizal fungi on insect herbivores: a meta-analysis. Ecology 90, 2088–2097. doi: 10.1890/08-1555.1 19739371

[B46] Kundu A. Mishra S. Vadassery J. (2018). Spodoptera litura-mediated chemical defense is differentially modulated in older and younger systemic leaves of solanum lycopersicum. Planta 248, 981–997. doi: 10.1007/s00425-018-2953-3 29987372

[B47] Lee Díaz A. S. Macheda D. Saha H. Ploll U. Orine D. Biere A. (2021). Tackling the context-dependency of microbial-induced resistance. Agronomy 11, 1293. doi: 10.3390/agronomy11071293

[B48] Livak K. J. Schmittgen T. D. (2001). Analysis of relative gene expression data using real-time quantitative PCR and the 2-ΔΔCT method. Methods 25, 402–408. doi: 10.1006/meth.2001.1262 11846609

[B49] Luo X. Li Z. Xiao S. Ye Z. Nie X. Zhang X.. (2021). Phosphate deficiency enhances cotton resistance to *Verticillium dahliae* through activating jasmonic acid biosynthesis and phenylpropanoid pathway. Plant Sci. 302, 110724. doi: 10.1016/j.plantsci.2020.110724 33288028

[B50] Machado R. A. Robert C. A. Arce C. C. Ferrieri A. P. Xu S. Jimenez-Aleman G. H.. (2016). Auxin is rapidly induced by herbivore attack and regulates a subset of systemic, jasmonate-dependent defenses. Plant Physiol. 172, 521–532. doi: 10.1104/pp.16.00940 27485882 PMC5074610

[B51] Major I. T. Yoshida Y. Campos M. L. Kapali G. Xin X. F. Sugimoto K.. (2017). Regulation of growth–defense balance by the JASMONATE ZIM-DOMAIN (JAZ)-MYC transcriptional module. New Phytol. 215, 1533–1547. doi: 10.1111/nph.14638 28649719 PMC5542871

[B52] Manresa-Grao M. Pastor-Fernández J. Sanchez-Bel P. Jaques J. A. Pastor V. Flors V. (2022). Mycorrhizal symbiosis triggers local resistance in citrus plants against spider mites. Front. Plant Sci. 13. doi: 10.3389/fpls.2022.867778 PMC9285983 35845655

[B53] Marro N. Lidoy J. Chico M.Á Rial C. García J. Varela R. M.. (2022). Strigolactones: New players in the nitrogen–phosphorus signalling interplay. Plant Cell Environ. 45, 512–527. doi: 10.1111/pce.14212 34719040

[B54] Martinez-Medina A. Flors V. Heil M. Mauch-Mani B. Pieterse C. M. Pozo M. J.. (2016). Recognizing plant defense priming. Trends Plant Sci. 21, 818–822. doi: 10.1016/j.tplants.2016.07.009 27507609

[B55] Mattson J. W.J. (1980). Herbivory in relation to plant nitrogen content. Annu. Rev. Ecol. Syst. 11, 119–161. doi: 10.1146/annurev.es.11.110180.001003

[B56] Mauch-Mani B. Bacelli I. Luna E. Flors V. (2017). Defense priming: an adaptative part of induced resistance. Annu. Rev. Plant Biol. 68, 485–512. doi: 10.1146/annurev-arplant-042916-041132 28226238

[B57] Mitra D. Djebaili R. Pellegrini M. Mahakur B. Sarker A. Chaudhary P.. (2021). Arbuscular mycorrhizal symbiosis: plant growth improvement and induction of resistance under stressful conditions. J. Plant Nutr. 44, 1993–2028. doi: 10.1080/01904167.2021.1881552

[B58] Mora-Romero G. A. Gonzalez-Ortiz M. A. Quiroz-Figueroa F. Calderon-Vazquez C. L. Medina-Godoy S. Maldonado-Mendoza I.. (2014). PvLOX2 silencing in common bean roots impairs arbuscular mycorrhiza-induced resistance without affecting symbiosis establishment. Funct. Plant Biol. 42 (1), 18–30. doi: 10.1071/FP14101 32480650

[B59] Orozco-Cárdenas M. L. Ryan C. A. (1999). Hydrogen peroxide is generated systemically in plant leaves by wounding and systemin *via* the octadecanoid pathway. PNAS 96 (11), 6553–6557. doi: 10.1073/pnas.96.11.6553 10339626 PMC26920

[B60] Perkins M. C. Woods H. A. Harrison J. F. Elser J. J. (2004). Dietary phosphorus affects the growth of larval *Manduca sexta* . Arch. Insect Biochem. Physiol. Behav. 55, 153–168. doi: 10.1002/arch.10133 14981659

[B61] Pieterse C. M. Zamioudis C. Berendsen R. L. Weller D. M. Van Wees S. C. Bakker P. A. (2014). Induced systemic resistance by beneficial microbes. Annu. Rev. Phytopathol. 52, 347–375. doi: 10.1146/annurev-phyto-082712-102340 24906124

[B62] Pozo M. J. Albrectsen B. R. Bejarano E. R. de la Pena E. Herrero S. Martinez-Medina A.. (2020). Three-way interactions between plants, microbes, and arthropods (PMA): impacts, mechanisms, and prospects for sustainable plant protection. Plant Cell 32, 1-11. doi: 10.1105/tpc.120.tt0720 32748801 PMC7346573

[B63] Pozo M. J. Azcón-Aguilar C. (2007). Unraveling mycorrhiza-induced resistance. Curr. Opin. Plant Biol. 10, 393–398. doi: 10.1016/j.pbi.2007.05.004 17658291

[B64] Pozo de la Hoz J. Rivero J. Azcón-Aguilar C. Urrestarazu M. Pozo M. J. (2021). Mycorrhiza-induced resistance against foliar pathogens is uncoupled of nutritional effects under different light intensities. J. Fungi 7, 402. doi: 10.3390/jof7060402 PMC8224078 34063889

[B65] Pozo M. J. López-Ráez J. A. Azcón-Aguilar C. García-Garrido J. M. (2015). Phytohormones as integrators of environmental signals in the regulation of mycorrhizal symbioses. New Phytol. 205, 1431–1436. doi: 10.1111/nph.13252 25580981

[B66] Pozo M. J. Zabalgogeazcoa I. de Aldana B. R. V. Martinez-Medina A. (2021). Untapping the potential of plant mycobiomes for applications in agriculture. Curr. Opin. Plant Biol. 60, 102034. doi: 10.1016/j.pbi.2021.102034 33827007

[B67] Pretty J. Bharucha Z. P. (2018). Sustainable intensification of agriculture: greening the world’s food economy. 1st edn (London: Routledge). doi: 10.4324/9781138638044

[B68] Qu L. Wang M. Biere A. (2021). Interactive effects of mycorrhizae, soil phosphorus, and light on growth and induction and priming of defense in *Plantago lanceolata* . Front. Plant Sci. 12. doi: 10.3389/fpls.2021.647372 PMC8021950 33833771

[B69] Rabino I. Mancinelli A. L. (1986). Light, temperature, and anthocyanin production. Plant Physiol. 81, 922–924. doi: 10.1104/pp.81.3.922 16664926 PMC1075451

[B70] Ramírez-Serrano B. Querejeta M. Minchev Z. Gamir J. Perdereau E. Pozo M. J.. (2022). Mycorrhizal benefits on plant growth and protection against *Spodoptera exigua* depend on n availability. J. Plant Interact. 17, 940–955. doi: 10.1080/17429145.2022.2120212

[B71] Rivero J. Álvarez D. Flors V. Azcón-Aguilar C. Pozo M. J. (2018). Root metabolic plasticity underlies functional diversity in mycorrhiza-enhanced stress tolerance in tomato. New Phytol. 220, 1322–1336. doi: 10.1111/nph.15295 29982997

[B72] Rivero J. Lidoy J. Llopis-Giménez A. Herrero S. Flors V. Pozo M. J. (2021). Mycorrhizal symbiosis primes the accumulation of antiherbivore compounds and enhances herbivore mortality in tomato. J. Exp. Bot. 72, 5038–5050. doi: 10.1093/jxb/erab171 33884424 PMC8219033

[B73] Rouached H. Arpat A. B. Poirier Y. (2010). Regulation of phosphate starvation responses in plants: signaling players and cross-talks. Mol. Plant 3, 288–299. doi: 10.1093/mp/ssp120 20142416

[B74] Salmeron-Santiago I. A. Martínez-Trujillo M. Valdez-Alarcón J. J. Pedraza-Santos M. E. Santoyo G. Pozo M. J.. (2021). An updated review on the modulation of carbon partitioning and allocation in arbuscular mycorrhizal plants. Microorganisms 10, 75. doi: 10.3390/microorganisms10010075 35056524 PMC8781679

[B75] Sanchez-Bel P. Sanmartín N. Pastor V. Mateu D. Cerezo M. Vidal-Albalat A.. (2018). Mycorrhizal tomato plants fine tunes the prowth-defence balance upon n depleted root enviroments. Plant Cell Environ. 41, 406–420. doi: 10.1111/pce.13105 29194658

[B76] Sanchez-Bel P. Troncho P. Gamir J. Pozo M. J. Camañes G. Cerezo M.. (2016). The nitrogen availability interferes with mycorrhiza-induced resistance against *Botrytis cinerea* in tomato. Front. Microbiol. 7, 1–16. doi: 10.389/fmicb.2016.01598 27790197 PMC5064179

[B77] Sanmartín N. Sánchez-Bel P. Pastor V. Pastor-Fernández J. Mateu D. Pozo M. J.. (2020). Root-to-shoot signalling in mycorrhizal tomato plants upon *Botrytis cinerea* infection. Plant Sci. 298, 110595. doi: 10.1016/j.plantsci.2020.110595 32771152

[B78] Schoenherr A. P. Rizzo E. Jackson N. Manosalva P. Gomez S. K. (2019). Mycorrhiza-induced resistance in potato involves priming of defense responses against cabbage looper (Noctuidae: Lepidoptera). Environ. Entomol. 48, 370–381. doi: 10.1093/ee/nvy195 30715218

[B79] Selvaraj A. Thangavel K. (2021). “Arbuscular mycorrhizal fungi: Potential plant protective agent against herbivorous insect and its importance in sustainable agriculture,” in Symbiotic soil microorganisms (Switzerland: Springer, Cham), 319–337. doi: 10.1007/978-3-030-51916-2_19

[B80] Selvaraj A. Thangavel K. Uthandi S. (2020). Arbuscular mycorrhizal fungi (*Glomus intraradices*) and diazotrophic bacterium (*Rhizobium* BMBS) primed defense in blackgram against herbivorous insect (*Spodoptera litura*) infestation. Microbiol. Res. 231, 126355. doi: 10.1016/j.micres.2019.126355 31704544

[B81] Shafiei F. Shahidi-Noghabi S. Sedaghati E. (2022). The impact of arbuscular mycorrhizal fungi on tomato plant resistance against *Tuta absoluta* (Meyrick) in greenhouse conditions. J. Asia-Pac. Entomol. 25, 101971. doi: 10.1016/j.aspen.2022.101971 38356097

[B82] Smakowska E. Kong J. Busch W. Belkhadir Y. (2016). Organ-specific regulation of growth-defense tradeoffs by plants. Curr. Opin. Plant Biol. 29, 129–137. doi: 10.1104/pp.111.174581 26802804

[B83] Smith S. E. Jakobsen I. Grønlund M. Smith F. A. (2011). Roles of arbuscular mycorrhizas in plant phosphorus nutrition: interactions between pathways of phosphorus uptake in arbuscular mycorrhizal roots have important implications for understanding and manipulating plant phosphorus acquisition. Plant Physiol. 156, 1050–1057. doi: 10.1104/pp.111.174581 21467213 PMC3135927

[B84] Smith S. E. Smith F. A. (2011). Roles of arbuscular mycorrhizas in plant nutrition and growth: new paradigms from cellular to ecosystem scales. Annu. Rev. Plant Biol. 62, 227–250. doi: 10.1146/annurev-arplant-042110-103846 21391813

[B85] Song Y. Chen D. Lu K. Sun Z. Zeng R. (2015). Enhanced tomato disease resistance primed by arbuscular mycorrhizal fungus. Front. Plant Sci. 6. doi: 10.3389/fpls.2015.00786 PMC4585261 26442091

[B86] Song Y. Y. Ye M. Li C. Y. Wang R. L. Wei X. C. Luo S. M.. (2013). Priming of anti-herbivore defense in tomato by arbuscular mycorrhizal fungus and involvement of the jasmonate pathway. J. Chem. Ecol. 39, 1036–1044. doi: 10.1007/s10886-013-0312-1 23797931

[B87] Treseder Kathleen K. (2013). The extent of mycorrhizal colonization of roots and its influence on plant growth and phosphorus content. Plant Soil 371, 1–13. doi: 10.1007/s11104-013-1681-5

[B88] Uppalapati S. R. Ayoubi P. Weng H. Palmer D. A. Mitchell R. E. Jones W.. (2005). The phytotoxin coronatine and methyl jasmonate impact multiple phytohormone pathways in tomato. Plant J. 42, 201–217. doi: 10.1111/j.1365-313X.2005.02366.x 15807783

[B89] Val-Torregrosa B. Bundó M. Martín-Cardoso H. Bach-Pages M. Chiou T. J. Flors V.. (2022). Phosphate-induced resistance to pathogen infection in *Arabidopsis* . Plant J. 110, 452–469. doi: 10.1111/tpj.15680 35061924 PMC9303409

[B90] van der Heyde M. Ohsowski B. Abbott L. K. Hart M. (2017). Arbuscular mycorrhizal fungus responses to disturbance are context-dependent. Mycorrhiza 27, 431–440. doi: 10.1007/s00572-016-0759-3 28120111

[B91] Vierheilig H. Schweiger P. Brundrett M. (2005). An overview of methods for the detection and observation of arbuscular mycorrhizal fungi in roots. Physiol. Plant 125, 393–404. doi: 10.1111/j.1399-3054.2005.00564.x

[B92] Vos I. A. Verhage A. Schuurink R. C. Watt L. G. Pieterse C. M. J. Van Wees S. C. M. (2013). Onset of herbivore-induced resistance in systemic tissue primed for jasmonate-dependent defenses is activated by abscisic acid. Front. Plant Sci. 4. doi: 10.3389/fpls.2013.00539 PMC3874679 24416038

[B93] Vysotskaya L. B. Trekozova A. W. Kudoyarova G. R. (2016). Effect of phosphorus starvation on hormone content and growth of barley plants. Acta Physiol. Plant 38, 1–6. doi: 10.1007/s11738-016-2127-5

[B94] Wang Z. Kuo H. F. Chiou T. J. (2021). Intracellular phosphate sensing and regulation of phosphate transport systems in plants. Plant Physiol. 187, 2043–2055. doi: 10.1093/plphys/kiab343 35235674 PMC8644344

[B95] Wang C. Tian B. Yu Z. Ding J. (2020). Effect of different combinations of phosphorus and nitrogen fertilization on arbuscular mycorrhizal fungi and aphids in wheat. Insects 11, 365. doi: 10.3390/insects11060365 32545401 PMC7349843

[B96] Wickham H. (2016). ggplot2: Elegant Graphics for Data Analysis (Verlag New York: Springer). Available at: https://ggplot2.tidyverse.org.

[B97] Yan L. Zhai Q. Wei J. Li S. Wang B. Huang T.. (2013). Role of tomato lipoxygenase d in wound-induced jasmonate biosynthesis and plant immunity to insect herbivores. PloS Genet. 9, e1003964. doi: 10.1371/journal.pgen.1003964 24348260 PMC3861047

[B98] Young J. P. W. (2015). Genome diversity in arbuscular mycorrhizal fungi. Curr. Opin. Plant Biol. 26, 113–119. doi: 10.1016/j.pbi.2015.06.005 26190590

[B99] Zeng M. Hause B. van Dam N. M. Uthe H. Hoffmann P. Krajinski F.. (2022). The mycorrhizal symbiosis alters the plant defense strategy in a model legume plant. Plant Cell Environ 45, 3412-3428. doi: 10.1111/pce.14421 35982608

