# Supplementary figures and images for: Phosphorus availability drives mycorrhiza induced resistance in tomato
Source: Front Plant Sci. 2022 Dec 19;13:1060926. doi: 10.3389/fpls.2022.1060926 (PMC9806178; doi:10.3389/fpls.2022.1060926)

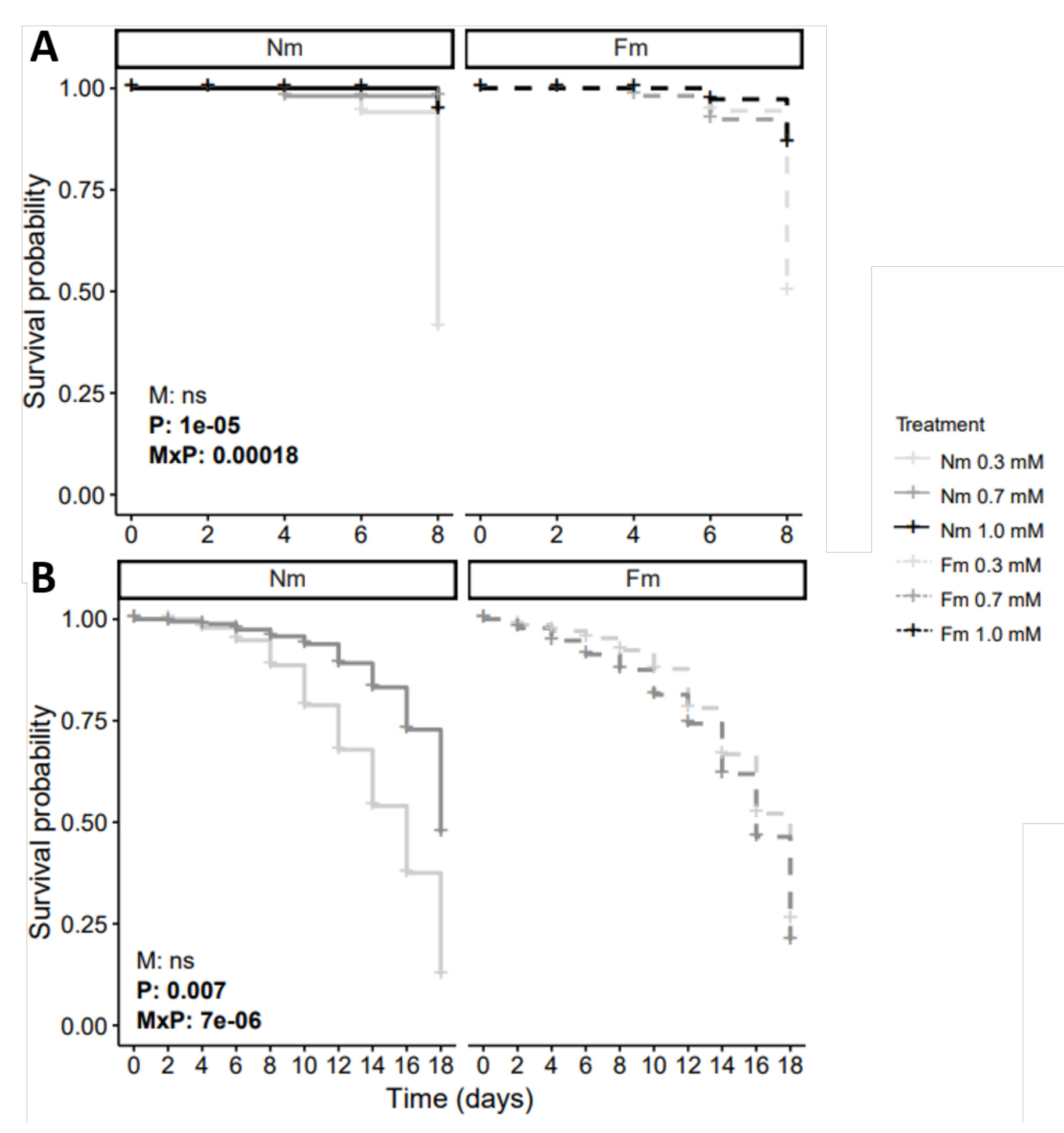

Supplement: Supplementary Figure 1 — Survival probability curves of S. exigua fed on leaves of mycorrhizal (Fm, dotted line) and non-mycorrhizal (Nm, continuous line) tomato plants. (A) Larvae were feeding on detached leaves from plants fertilized with 0.3 (light grey), 0.7 (grey) or 1.0 (black) mM of H2NaPO₄, one leaf of each plant (n=10) was detached 8 weeks pmi and infested with two second instar S. exigua larvae (n=20 larvae). (B) Larvae feeding on leaves of plants (n=9) fertilized with 0.3 mM (light grey) or 0.7 mM (grey) of H2NaPO₄. Two second instar larvae were added to one leaf of intact plants using clip-cages (n=18 per treatment) 6 weeks pmi, and infestation was maintained for three weeks more. Survival distribution comparisons were performed using the non-parametric Log-rank (Mantel-Cox) test. [file Image_1.jpeg]

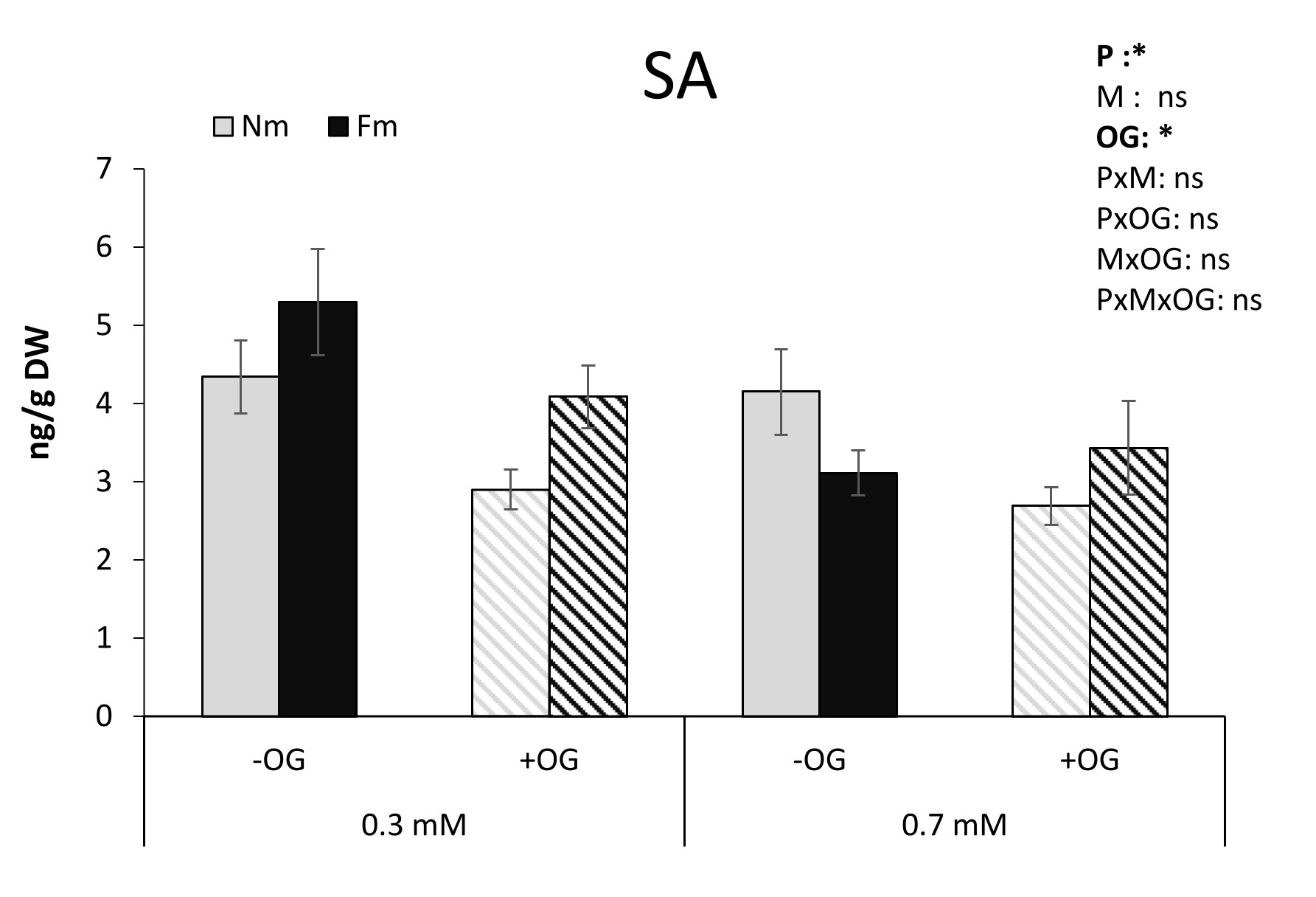

Supplement: Supplementary Figure 2 — Salicylic acid content in non-mycorrhizal (Nm) and mycorrhizal tomato plants colonized by F. mosseae (Fm) grown under 0.3 mM or 0.7 mM P fertilization, and treated with 50 μg/ml OG solution (+OG; stripped bars) or water as control (-OG; filled bars), harvested 6h post elicitation. Multiway ANOVA using P fertilization (P) mycorrhizal (M) and OG treatment (OG) as factors was performed and significance is indicated in the upper right corner of the graph. Asterisks denote significant effect of a factor or their interaction. ns: no significant; *: p<0.05; **: p<0.01; ***: p<0.001; ****: p<0.0001. [file Image_2.jpeg]
